# Genomic evolution of *Neisseria gonorrhoeae* since the preantibiotic era (1928–2013): antimicrobial use/misuse selects for resistance and drives evolution

**DOI:** 10.1186/s12864-020-6511-6

**Published:** 2020-02-03

**Authors:** Daniel Golparian, Simon R. Harris, Leonor Sánchez-Busó, Steen Hoffmann, William M. Shafer, Stephen D. Bentley, Jörgen S. Jensen, Magnus Unemo

**Affiliations:** 1 WHO Collaborating Centre for Gonorrhoea and other Sexually Transmitted Infections, Department of Laboratory Medicine, Microbiology, Faculty of Medicine and Health, Örebro University, SE-710 85 Örebro, Sweden; 2 Microbiotica Ltd, Biodata Innovation Centre, Wellcome Genome Campus, Hinxton, Cambridgeshire, UK; 3 Centre for Genomic Pathogen Surveillance, Wellcome Sanger Institute, Wellcome Genome Campus, Hinxton, Cambridgeshire, UK; 4 Big Data Institute, Nuffield Department of Medicine, University of Oxford, Oxford, UK; 5 Infection Preparedness, Research Unit for Reproductive Tract Microbiology, Statens Serum Institut, Copenhagen, Denmark; 6 Department of Microbiology and Immunology and Emory Antibiotic Resistance Center, Emory University School of Medicine, Atlanta, GA USA; 7 Laboratories of Bacterial Pathogenesis, VA Medical Center, Decatur, GA USA; 8 Pathogen Genomics, Wellcome Sanger Institute, Wellcome Genome Campus, Hinxton, Cambridgeshire, UK

**Keywords:** *Neisseria gonorrhoeae*, Antimicrobial resistance, Evolution, Whole-genome sequencing, Genomic epidemiology, Temporal analysis

## Abstract

**Background:**

Multidrug-resistant *Neisseria gonorrhoeae* strains are prevalent, threatening gonorrhoea treatment globally, and understanding of emergence, evolution, and spread of antimicrobial resistance (AMR) in gonococci remains limited. We describe the genomic evolution of gonococci and their AMR, related to the introduction of antimicrobial therapies, examining isolates from 1928 (preantibiotic era) to 2013 in Denmark. This is, to our knowledge, the oldest gonococcal collection globally.

**Methods:**

Lyophilised isolates were revived and examined using Etest (18 antimicrobials) and whole-genome sequencing (WGS). Quality-assured genome sequences were obtained for 191 viable and 40 non-viable isolates and analysed with multiple phylogenomic approaches.

**Results:**

Gonococcal AMR, including an accumulation of multiple AMR determinants, started to emerge particularly in the 1950s–1970s. By the twenty-first century, resistance to most antimicrobials was common. Despite that some AMR determinants affect many physiological functions and fitness, AMR determinants were mainly selected by the use/misuse of gonorrhoea therapeutic antimicrobials. Most AMR developed in strains belonging to one multidrug-resistant (MDR) clade with close to three times higher genomic mutation rate. Modern *N. gonorrhoeae* was inferred to have emerged in the late-1500s and its genome became increasingly conserved over time.

**Conclusions:**

WGS of gonococci from 1928 to 2013 showed that no AMR determinants, except *penB*, were in detectable frequency before the introduction of gonorrhoea therapeutic antimicrobials. The modern gonococcus is substantially younger than previously hypothesized and has been evolving into a more clonal species, driven by the use/misuse of antimicrobials. The MDR gonococcal clade should be further investigated for early detection of strains with predispositions to develop and maintain MDR and for initiation of public health interventions.

## Background

The twentieth century witnessed enormous successes in antimicrobial treatment for infectious diseases, drastically reducing the burden of diseases, especially from the mid-1940s to 1970s, named the golden era of antimicrobial discovery [1]. However, particularly in the 1980s and early 1990s, antimicrobial resistance (AMR) began to substantially threaten treatment and control of many infectious diseases, driving the development and introduction of new antimicrobials. Nevertheless, pathogens have continued to evolve AMR mechanisms faster than new therapies have been developed and only few antimicrobials with novel mechanisms of action have been developed during the twenty-first century [2].

The sexually transmitted infection gonorrhoea is a global public health problem and in 2016, the World Health Organization (WHO) estimated 87 million new cases each year globally [3]. *Neisseria gonorrhoeae* (gonococcus), the causative agent of gonorrhoea, can effectively illustrate how a bacterial pathogen can develop AMR to all classes of therapeutic antimicrobials. *N. gonorrhoeae* has evolved (developed or acquired), and maintained resistance to all drugs introduced for treatment of gonorrhoea since the discovery of antimicrobials [4]. This AMR evolution has spanned from the international introduction of sulfonamides in the late-1930s-1940s to the currently recommended extended-spectrum cephalosporins (ESCs), including an increase in multidrug-resistant (MDR) strains, during the past decade [4, 5]. The gonococcus has used basically all known physiological mechanisms of AMR such as i) antimicrobial degradation or modification by enzymes, ii) antimicrobial target modification or protection, iii) reduced antimicrobial influx, and iv) increased antimicrobial efflux [4]. The AMR determinants have evolved through selection or acquisition of chromosomal mutations or AMR-mediating plasmids. *N. gonorrhoeae* has an extraordinary ability to change its genome as a response to external pressure through mutations and horizontal gene transfer (whole or partial genes), especially because it is naturally competent for transformation during its entire life cycle [4, 6].

In the last 1–2 decade(s), there have been worrying reports from many continents of gonococcal strains with resistance to the ESCs, which are the last remaining alternatives for empiric first-line monotherapy of gonorrhoea [4, 5, 7–10]. As a consequence, WHO global gonorrhoea treatment guidelines and guidelines in Europe, Australia, USA, and Canada currently recommend dual antimicrobial therapy, mostly ceftriaxone 250–500 mg single intramuscular dose plus azithromycin 1–2 g single oral dose, while some countries such as Japan and the UK since 2019 recommends only high-dose (1 g) ceftriaxone monotherapy [9, 11]. Unfortunately, sporadic gonococcal isolates with ceftriaxone resistance have been described in many countries, and azithromycin resistance has been described in most settings worldwide [4, 5, 7–13], and the first global failure with ceftriaxone plus azithromycin therapy was verified in the UK [12]. Furthermore, international transmission of the ceftriaxone-resistant strain FC428 was verified in 2015–2019 [7, 10, 13, 14] and in 2018 the first strain with ceftriaxone resistance combined with high-level azithromycin resistance was reported in the UK and Australia [8]. This AMR evolution strongly emphasises the urgent need to significantly improve our understanding of the emergence (selection), evolution and spread of AMR *N. gonorrhoeae*. The knowledge regarding many of these unknowns can only really effectively be increased by investigating *N. gonorrhoeae* and its AMR emergence and evolution during the entire antimicrobial era, including when and how AMR emerged and how it was maintained without significant fitness loss.

Whole-genome sequencing (WGS) provides the ideal means to study in-depth the mechanisms that promote AMR emergence, evolution and spread, tracking pathogen populations with unprecedented sensitivity for genetic variation and greatly improved resolution for studies in population dynamics. As a result, WGS is driving the understanding of regulatory networks, transmission and AMR in the gonococcus [15–21].

Herein, we describe the genomic evolution of *N. gonorrhoeae* and its AMR, in relation to the introduction of antimicrobials for gonorrhoea therapy, in a unique collection of isolates from 1928 (preantibiotic era) to 2013 in Denmark. This is, to our knowledge, the oldest collection of gonococcal isolates globally, which was preserved at the Statens Serum Institut (SSI), Copenhagen, Denmark, that has been operating nationally under the Danish Ministry of Health for more than 100 years.

## Methods

### Biological specimens

Out of 617 lyophilised *N. gonorrhoeae* isolates received from the SSI, Copenhagen, Denmark, 191 *N. gonorrhoeae* isolates, species-confirmed using MALDI-TOF-MS (Bruker Daltonics, Bremen, Germany), from 1928 to 2013 were revived. The isolates from 2000 to 2013 were randomly selected based on different antimicrobial phenotypes and year of isolation. To expand the number of examined isolates cultured prior to the 1970s, we additionally included 81 non-viable isolates for WGS (Additional file 2: Figure S1).

### Laboratory procedures

Minimum inhibitory concentrations (MICs; mg/L) were determined using Etest (bioMérieux, Marcy-l’Étoile, France), as previously described [19], for all antimicrobials previously or currently recommended and/or used for the treatment of gonorrhoea internationally and several additional antimicrobials of interest (*n* = 18). MICs were interpreted using breakpoints for susceptibility (S) and resistance (R) according to the European Committee on Antimicrobial Susceptibility Testing (EUCAST; www.eucast.org) where available (Additional file 1).

Genomic DNA was isolated using the Wizard Genomic DNA Purification Kit (Promega Corporation, Madison, WI, USA), according to the manufacturer’s instructions with minor modifications (Additional file 1). DNA from the 81 non-viable isolates was amplified using the illustra Genomiphi V2 kit (GE Healthcare, Little Chalfont, UK) and an in-house *N. gonorrhoeae porA* pseudogene PCR performed to confirm the presence of *N. gonorrhoeae* DNA [22], prior to WGS. WGS was performed with multiplex libraries with 100 base paired-end reads using Illumina HiSeq 4000 at the Wellcome Sanger Institute, Cambridge, UK.

### Bioinformatic analysis

The quality controls of all WGS data, assembly, characterization of the resistome (known AMR determinants [4, 23, 24]) and molecular epidemiology, including *N. gonorrhoeae* multi-antigen sequence typing (NG-MAST), multi-locus sequence typing (MLST), and *N. gonorrhoeae* Sequence Typing for Antimicrobial Resistance (NG-STAR), single-nucleotide polymorphism (SNP)-based phylogenomics, temporal dating analysis, and methodology for defining core genomes are detailed in the Additional file 1.

## Results

### Evolution of antimicrobial resistance and resistance determinants in *N. gonorrhoeae* specimens from Denmark in 1928–2013

The 231 samples were divided into three distinct eras: “preantibiotic” (pre-1950s), “golden” (1950–1970s), and “postmodern” (1980-twenty-first century). All viable isolates (*n* = 191) were tested against 18 antimicrobials, which included those currently and previously used against gonorrhoea as well as some additional antimicrobials (Table 1).

**Table 1:** Antimicrobial resistance and resistance determinants for *Neisseria gonorrhoeae* isolates from Denmark, 1928–2013

Antibiotic	Preantibiotic era (% S/I/R)			Golden era (% S/I/R)			Postmodern era (% S/I/R)		
	1920s (*n* = 3)	1930s (*n* = 11)	1940s (*n* = 41)	1950s (*n* = 37)	1960s (*n* = 27)	1970s (*n* = 10)	1980s (*n* = 15)	1990s (*n* = 14)	21st century (*n* = 33)
Penicillin G	100.0/−/−	100.0/−/−	100.0/−/−	91.9/8.1/−	55.6/40.7/3.7	80.0/10.0/10.0	40.0/53.3/6.7	14.3/50.0/35.7	6.1/48.5/45.4
TMP-SMX	100.0/−/−	100.0/−/−	53.7/43.9/2.4	59.5/37.8/2.7	40.8/48.1/11.1	50.0/40.0/10.0	53.3/26.7/20.0	14.3/35.7/50.0	12.1/18.2/69.7
Chloramphenicol	100.0/−/−	90.9/−/9.1	100.0/−/−	100.0/−/−	77.8/18.5/3.7	80.0/10.0/10.0	73.3/6.7/20.0	35.7/21.4/42.9	24.2/48.5/27.3
Tetracycline	100.0/−/−	100.0/−/−	100.0/−/−	100.0/−/−	81.5/14.8/3.7	80.0/10.0/10.0	66.7/13.3/20.0	14.3/21.4/64.3	15.2/24.2/60.6
Ciprofloxacin	100.0/−/−	100.0/−/−	100.0/−/−	100.0/−/−	100.0/−/−	100.0/−/−	100.0/−/−	85.7/−/14.3	30.3/−/69.7
Erythromycin	100.0/NA/−	90.9/NA/9.1	100.0/NA/−	100.0/NA/−	92.6/NA/7.4	80.0/NA/20.0	73.3/NA/26.7	42.9/NA/57.1	42.4/NA/57.6
Azithromycin	100.0/NA/−	100.0/NA/−	100.0/NA/−	100.0/NA/−	100.0/NA/−	100.0/NA/−	100.0/NA/−	100.0/NA/−	90.9/NA/9.1
Cefuroxime	100.0/−/−	100.0/−/−	100.0/−/−	100.0/−/−	100.0/−/−	100.0/−/−	100.0/−/−	78.6/14.3/7.1	81.8/3.0/15.2
Cefotaxime	100.0/NA/−	100.0/NA/−	100.0/NA/−	100.0/NA/−	100.0/NA/−	100.0/NA/−	100.0/NA/−	92.9/NA/7.1	84.8/NA/15.2
Cefixime	100.0/NA/−	100.0/NA/−	100.0/NA/−	100.0/NA/−	100.0/NA/−	100.0/NA/−	100.0/NA/−	100.0/NA/−	97.0/NA/3.0
Ceftriaxone	100.0/NA/−	100.0/NA/−	100.0/NA/−	100.0/NA/−	100.0/NA/−	100.0/NA/−	100.0/NA/−	100.0/NA/−	100.0/NA/−
Spectinomycin	100.0/NA/−	100.0/NA/−	100.0/NA/−	100.0/NA/−	100.0/NA/−	100.0/NA/−	100.0/NA/−	100.0/NA/−	100.0/NA/−
Gentamicin	100.0/−/−	100.0/−/−	100.0/−/−	100.0/−/−	100.0/−/−	100.0/−/−	100.0/−/−	100.0/−/−	100.0/−/−
Kanamycin	100.0/−/−	100.0/−/−	100.0/−/−	100.0/−/−	100.0/−/−	100.0/−/−	93.3/6.7/−	92.9/7.1/−	100.0/−/−
Lower/medium/upper range (%)									
Sulfamethoxazole	100.0/−/−	100.0/−/−	63.4/26.8/9.8	83.8/8.1/8.1	74.1/18.5/7.4	80.0/10.0/10.0	66.7/13.3/20.0	14.3/21.4/64.3	12.1/30.3/57.6
Ampicillin	100.0/−/−	100.0/−/−	100.0/−/−	100.0/−/−	100.0/−/−	100.0/−/−	93.3/−/6.7	92.9/−/7.1	66.7/27.3/6.0
Rifampin	100.0/−/−	100.0/−/−	100.0/−/−	100.0/−/−	100.0/−/−	100.0/−/−	100.0/−/−	78.6/−/21.4	51.5/−/48.5
Ertapenem	100.0/−/−	100.0/−/−	100.0/−/−	100.0/−/−	100.0/−/−	100.0/−/−	100.0/−/−	100.0/−/−	100.0/−/−
Absence/presence of resistance mutations (affected drug or effect) (%)									
*penA* D345a (penicillin)	100.0/−	100.0/−	100.0/−	91.9/8.1	51.9/48.1	80.0/20.0	33.3/66.6	14.3/85.7	18.2/81.8
*penA* mosaic (penicillin, ESCs)	100.0/−	100.0/−	100.0/−	100.0/−	100.0/−	100.0/−	100.0/−	100.0/−	84.8/15.2
*penB* (decreased influx)	66.6/33.3	100.0/−	100.0/−	100.0/−	96.3/3.7	90.0/10.0	80.0/20.0	57.1/42.9	42.4/57.6
*mtrR* (increased efflux)	100.0/−	100.0/−	100.0/−	100.0/−	59.3/40.7	80.0/20.0	73.3/26.7	35.7/64.3	60.6/39.4
*gyrA* (ciprofloxacin)	100.0/−	100.0/−	95.1/4.9	97.3/2.7	100.0/−	100.0/−	100.0/−	85.7/14.3	33.3/66.7
*parC* (ciprofloxacin)	100.0/−	100.0/−	100.0/−	100.0/−	100.0/−	100.0/−	100.0/−	92.9/7.1	39.4/60.6
*ponA* (penicillin)	100.0/−	100.0/−	100.0/−	100.0/−	96.3/3.7	80.0/20.0	86.7/13.3	57.1/42.9	39.4/60.6
*folP* (sulfonamides)	100.0/−	100.0/−	97.6/2.4	100.0/−	96.3/3.7	80.0/20.0	80.0/20.0	21.4/78.6	12.1/87.9
*rpsJ* (tetracycline)	100.0/−	100.0/−	100.0/−	100.0/−	81.5/18.5	80.0/20.0	66.7/33.3	7.1/92.9	15.2/84.8
*tetM* plasmid (tetracycline)	100.0/−	100.0/−	100.0/−	100.0/−	100.0/−	100.0/−	100.0/−	71.4/28.6	69.7/30.3
*bla*_TEM_ (penicillin)	100.0/−	100.0/−	100.0/−	100.0/−	100.0/−	100.0/−	93.3/6.7	92.9/7.1	69.7/30.3
*rpsE* (spectinomycin)	100.0/−	100.0/−	100.0/−	100.0/−	100.0/−	100.0/−	100.0/−	100.0/−	100.0/−
16S rDNA (spectinomycin)	100.0/−	100.0/−	100.0/−	100.0/−	100.0/−	100.0/−	100.0/−	100.0/−	100.0/−
23S rDNA (azithromycin)	100.0/−	100.0/−	100.0/−	100.0/−	100.0/−	100.0/−	100.0/−	100.0/−	100.0/−
*rpoB* (rifampin)	100.0/−	100.0/−	100.0/−	100.0/−	100.0/−	100.0/−	100.0/−	78.6/21.4	51.5/48.5

*TMP-SMX* Trimethoprim-Sulfamethoxazole; *ESCs* Extended-spectrum cephalosporins; *NA* Not applicable

**Fig. 1:**
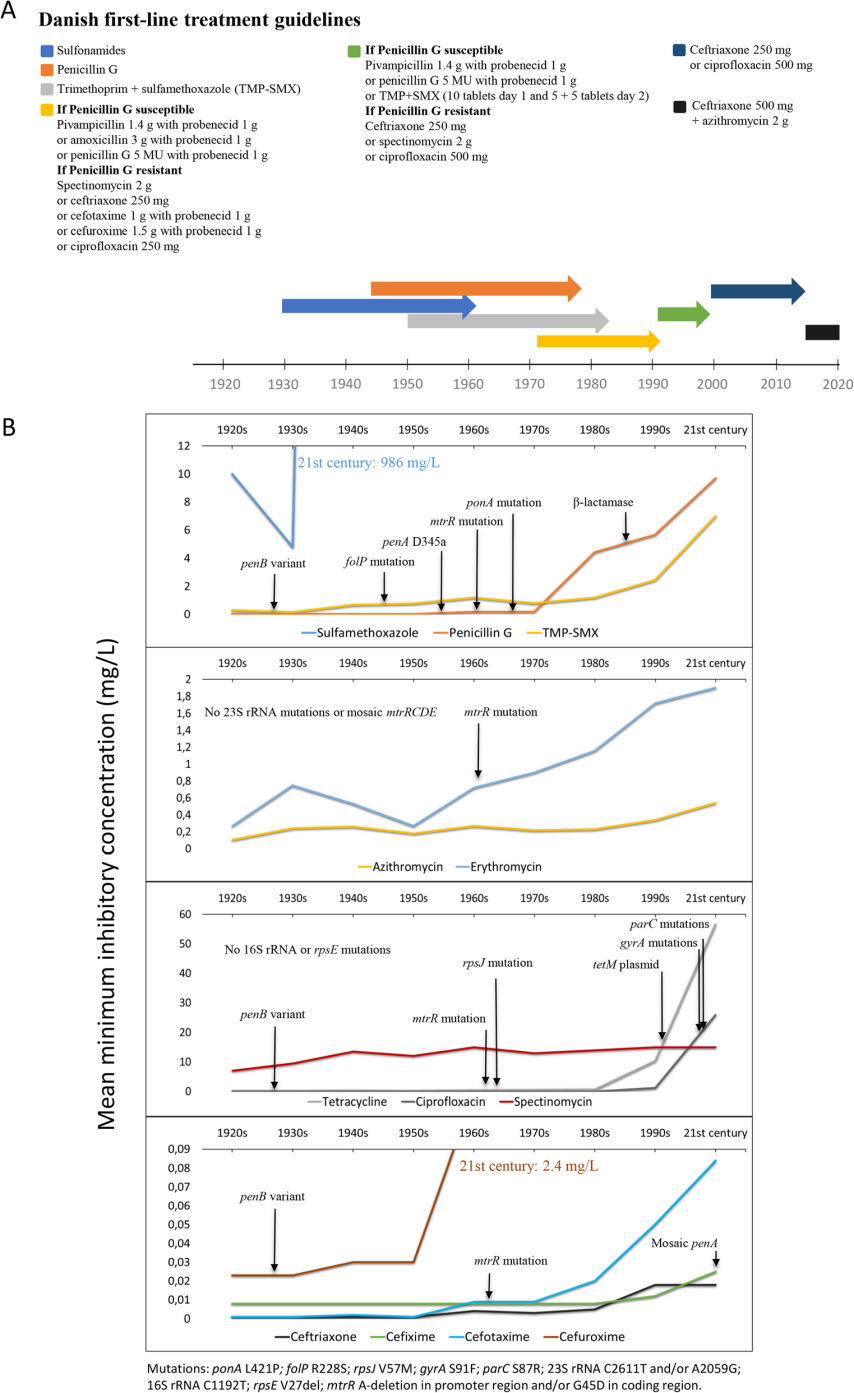
Treatment guidelines and antimicrobial resistance development in Denmark since the 1920s. **a** Coloured arrows indicate the time period the different antimicrobials were recommended and used for treatment of gonorrhoea in Denmark. **b** Mean MIC of antimicrobials previously and currently used for treatment of gonorrhoea (2–4 antimicrobials per box) and first identification of antimicrobial resistance determinants for the specific antimicrobials in the examined isolates from Denmark (black arrow)

As expected, decreased susceptibility or resistance was exceedingly rare in the preantibiotic era and the only case before the 1940s was one isolate cultured in 1930 with resistance to erythromycin (MIC = 4 mg/L). Notably, the azithromycin MIC for this isolate was exactly at the EUCAST ECOFF for azithromycin (1 mg/L), which is considered as a resistance breakpoint for azithromycin and erythromycin in the present study (Table 1). During the 1940s, decreased susceptibility to erythromycin (MIC 0.5–1 mg/L) continued to evolve without any known tested macrolide resistance determinant being detected. This was also the case for sulfamethoxazole (MIC > 256 mg/L found in 1942) and TMP-SMX (resistant isolates in 1946), though the decreased susceptibility and resistance was most likely due to the widespread use of other sulfonamides for treatment of gonorrhoea during this time period in contrast to erythromycin or other macrolides (Fig. 1). Five isolates from the 1940–1950s had a GyrA S91T amino acid substitution in the active site for fluoroquinolones, however, all five isolates were susceptible to ciprofloxacin. The drift towards higher MICs of macrolides continued during the golden era (1950–1970) with up to 20.0% of isolates resistant to erythromycin in the 1970s. Moreover, resistance or MICs in the upper range to penicillin G (0–10.0% in these decades), sulfamethoxazole (7.4–10.0%), TMP-SMX (2.7–11.1%), chloramphenicol (3.7–10.0%) and tetracycline (0–10.0%) started to emerge during the golden era (Table 1), concurrently with the identification of the first isolates harbouring chromosomal AMR determinants to penicillin (*penA* D345a in 1956 and *ponA1* (L421P) mutation in 1967), sulfonamides (*folP* R228S*,* 1946), and tetracycline (*rpsJ* V57 M*,* 1963), as well as a higher proportions of isolates with MtrCDE efflux pump mutations reducing the susceptibility to many antimicrobials (MtrR G45D (1961), *mtrR* A-deletion (1967), *mtr*_120_ (1982), MtrR G45S (2013)) (Table 1). The emergence of phenotypic AMR and detection of genetic AMR determinants happened primarily in isolates from the postmodern era. Among isolates from the 1980s, 6.7% were resistant or in the upper MIC range for penicillin G, ampicillin, and kanamycin. Furthermore, resistance or in the upper MIC range for sulfamethoxazole (20%), TMP-SMX (20%), chloramphenicol (20%), tetracycline (20%), and erythromycin (26.7%) was common (Table 1). In isolates from 1986 and the 1990s, the first β-lactamase and *tetM*-carrying plasmids were detected, respectively (Fig. 1), and consequently, the first isolates with high-level resistance to penicillins and tetracyclines. Isolates resistant to or in the upper MIC range for sulfamethoxazole, TMP-SMX, chloramphenicol, penicillin G, tetracycline, and erythromycin were very common (> 30%), and resistance to cephalosporins, such as cefuroxime (*n* = 1) and cefotaxime (n = 1), emerged during the 1990s (Table 1). During the twenty-first century, resistance or upper range MICs for sulfamethoxazole, penicillin G, TMP-SMX, tetracycline, erythromycin, rifampin, and ciprofloxacin was very common and for ampicillin, chloramphenicol, azithromycin, cefuroxime, and cefotaxime relatively common (6.1–27.3%). Furthermore, resistance to cefixime (3.0%) emerged in the twenty-first century (Table 1). Known ESC resistance determinants such as the mosaic *penA* (*penA-*34; 2009) and for fluoroquinolones, i.e. fluoroquinolone resistance-mediating *gyrA* S91F (1997) and *parC* S87R (1998) mutations, were detected for the first time in isolates from the postmodern era (Fig. 1). No viable isolate displayed resistance or upper MIC range for ceftriaxone, spectinomycin, gentamicin, or ertapenem.

Regarding AMR determinants affecting the susceptibility to many antimicrobials, interestingly, the first isolate with a *penB* variant (G120D), potentially causing a reduced intake of antimicrobials through the outer membrane PorB, was found in an isolate from 1928, i.e., before the introduction of penicillin or other therapeutic antimicrobials. Concerning AMR determinants increasing the efflux of many antimicrobials through the MtrCDE efflux pump, a mutation in *mtrR* (MtrR G45D) was detected in 1961, however, no isolate contained any *mtrD* or *mtrR* (including the promoter) mosaic gene [23, 24]. Notably, two isolates from 1980 and 2013, respectively, had a G70D mutation in a region of the L4 protein, encoded by *rplD*, associated with decreased susceptibility or resistance to macrolides, and both isolates were resistant to erythromycin but susceptible to azithromycin [18]. Finally, the first *N. gonorrhoeae* strain carrying parts of the human L1 element [25] was cultured already in this collection in 1946.

### Phylogenomics of *Neisseria gonorrhoeae* specimens from Denmark in 1928–2013

**Fig. 2:**
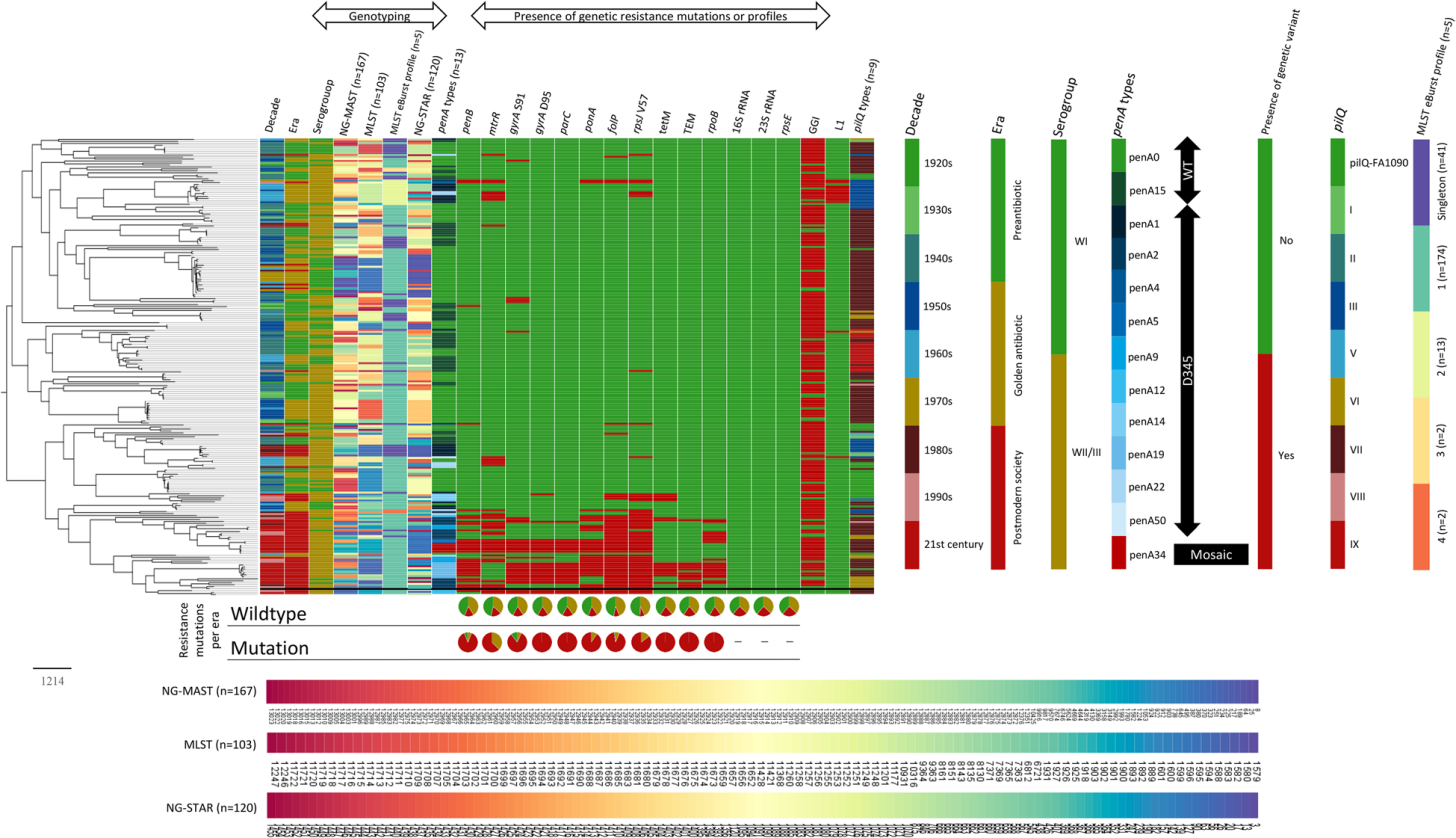
Core single nucleotide polymorphisms (SNPs) in 232 *Neisseria gonorrhoeae* genomes was initially determined using Gubbins to exclude the recombinant regions and a maximum likelihood tree was constructed using 28,196 SNPs. The columns next to the tree describe the antimicrobial resistance determinants in each isolate. The reference WHO O genome [19] is not annotated and is shown in black. More than 100 different *N. gonorrhoeae* multi-antigen sequence typing (NG-MAST), multilocus sequence typing (MLST), and *N. gonorrhoeae* sequence typing antimicrobial resistance (NG-STAR) sequence types were found. The pie charts below the tree reflect the proportion of each antimicrobial resistance determinant per era

Vertically-inherited SNPs resulting from mapping were used to assess the relationship between the 231 samples from nearly a century (Fig. 2). The analysis of non-recombinant SNPs increases the accuracy of the phylogenomic reconstruction and allows calculating the substitution rate of the organism without the effect of recombination. The phylogenomic tree had a high diversity with the most recent isolates being grouped into one main clade, the majority of these were from the postmodern era and carried the highest proportion of the known AMR determinants (Fig. 2). The high diversity of the isolates was also noticeable in the number of different NG-MAST sequence types (STs) (*n* = 167), NG-STAR STs (*n* = 120), and MLST STs (*n* = 103) (Fig. 2).

**Fig. 3:**
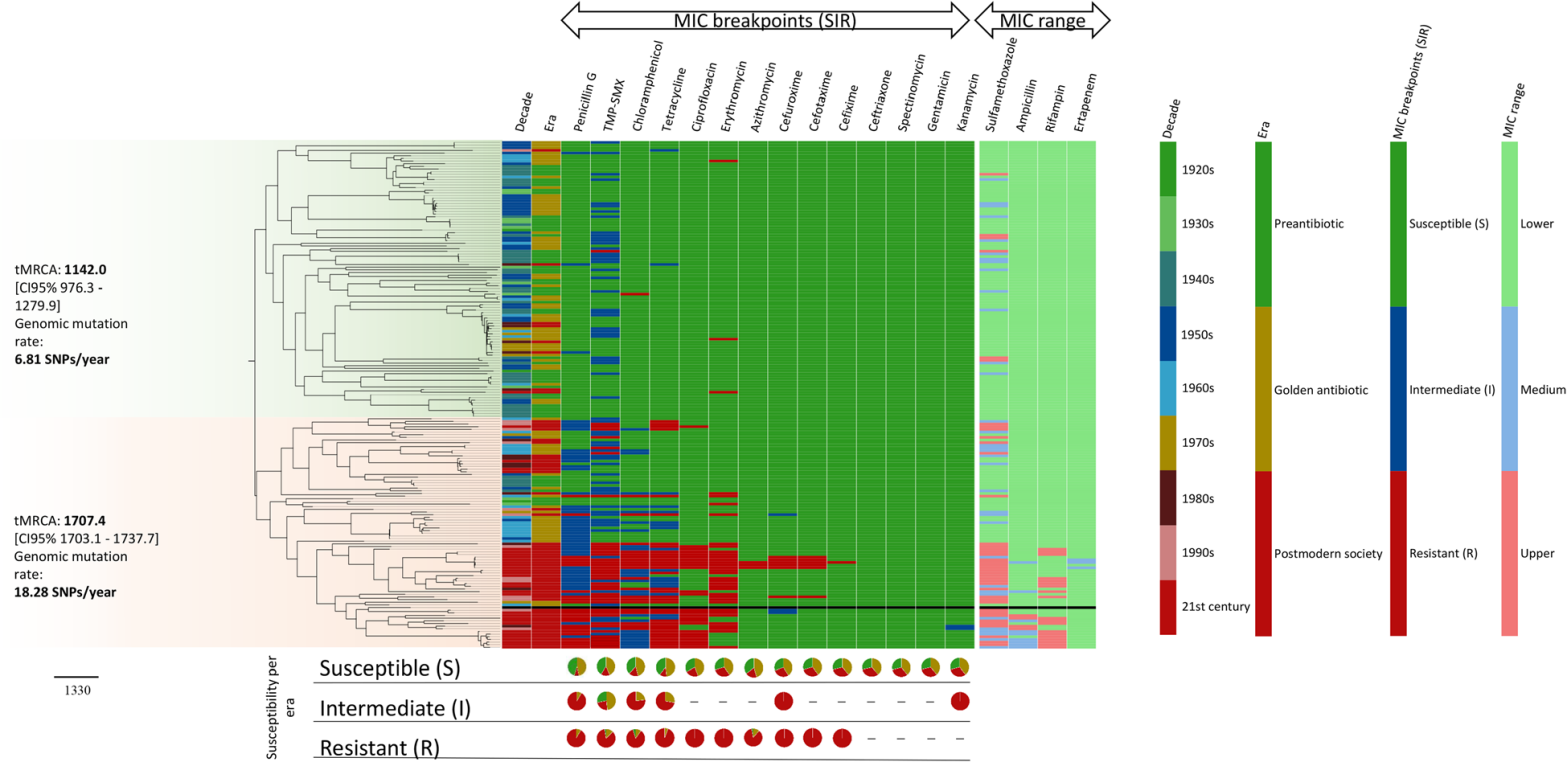
Core single nucleotide polymorphisms (SNPs) in 192 *Neisseria gonorrhoeae* genomes was initially obtained using Gubbins to exclude the recombinant regions and maximum likelihood tree was reconstructed using 26,744 SNPs. The columns next to the tree describe the antimicrobial susceptibility of each isolate. The antimicrobial susceptible clade is coloured in green and the multidrug-resistant clade in red. The pie charts below the tree reflect the proportion of isolates resistant to each antimicrobial per era. Reference strain WHO O [19] is included in the tree

The phylogenomic tree with exclusively the viable *N. gonorrhoeae* isolates (*n* = 191) was even more clearly divided into two main clades (Fig. 3), one consisted of antimicrobial susceptible isolates from the preantibiotic and the golden era, and the other one included mainly isolates from the postmodern era and the majority of isolates with AMR or decreased susceptibility to the tested antimicrobials, i.e. an MDR clade (Fig. 3). The smaller subclade with isolates from the twenty-first century displayed an antibiogram comparable to isolates from other AMR surveillance reports during the recent decade [26, 27]. Most isolates with decreased susceptibility or resistance to the tested antimicrobials were clustered together in the MDR clade with exception of erythromycin and sulfamethoxazole, where these isolates were unexpectedly spread across the tree, i.e. even in isolates cultured before the discovery of macrolides (although mainly in the MDR clade). Finally, we found isolates carrying gonococcal genetic island (GGIs) from 1928 to 2013, i.e. across all nine decades, with no association with antimicrobial exposure or AMR phenotype (Fig. 2).

### Core genome and pangenome of *Neisseria gonorrhoeae* specimens from Denmark in 1928–2013

**Fig. 4:**
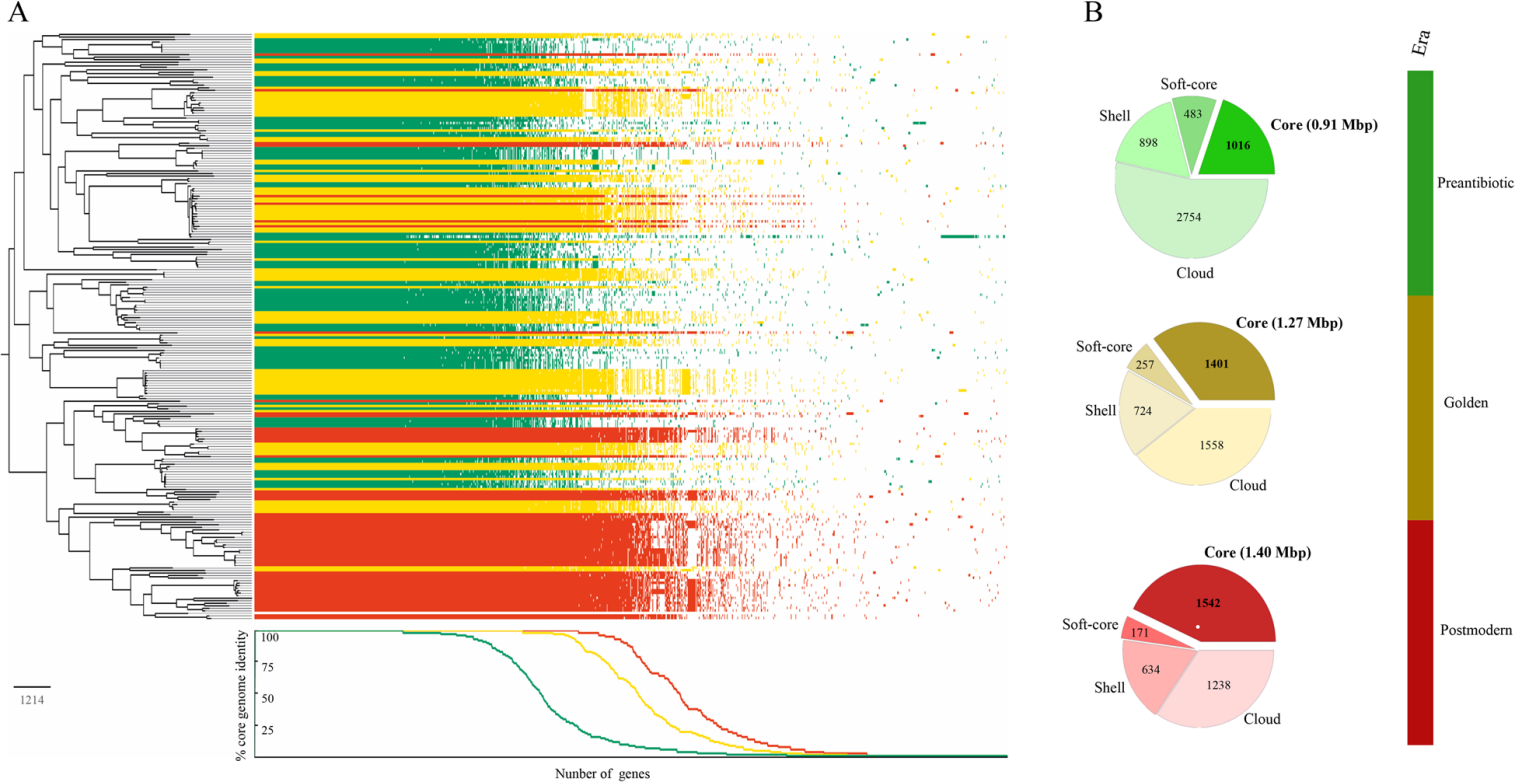
Phylogenomic tree with the size of the core genome for the three separate eras. **a** Phylogenomic tree including the size of the core genome for the preantibiotic, golden, and postmodern era consisting of 1016, 1401, and 1542 genes, respectively, using the Roary pan genome pipeline. **b** The proportion of the genes for each era is divided into core, soft-core, shell, and cloud genes, which are changing over time and the core genome length is increasing from 0.91 Mbp to 1.4 Mbp. Core = ≥99–100% of isolates share genes. Soft-core = ≥95- < 99% of isolates share genes. Shell = ≥15- < 95% of isolates share genes. Cloud = 0- < 15% of isolates share genes

The size of the core genome of the whole dataset (*n* = 231) and the comparison of the core genome (genes identified in ≥99% of the examined isolates) between the three antibiotic eras might be able to elucidate why certain clades are more successful. The core genome for all isolates (*n* = 231) included 1242 genes, with a pangenome of 6304 genes across all nine decades (Fig. 4, Additional file 2: Figure S2). However, the total number of core genes and thus, core genome length varied across the eras and appeared to increase over time, which likely illustrates that the whole genome becomes more conserved over time. Isolates belonging to the preantibiotic era had a smaller core genome, 1016 genes and a length of 0.91 Mbp, while the isolates in the golden era had a core genome of 1401 genes and a length of 1.27 Mbp. Finally, the most recent isolates from the postmodern era had the largest core genome including 1542 genes and 1.4 Mbp. In contrast, the size of the pangenome decreased over time (Fig. 4b). Furthermore, the nucleotide diversity of the common core genome of the isolates decreased over time and was 0.002644 (15,885 polymorphisms), 0.002634 (14,253 polymorphisms), and 0.002466 (12,862 polymorphisms) for the preantibiotic, golden, and postmodern era, respectively. The difference in diversity was most noticeably between the preantibiotic era and the postmodern era with a ratio of 1.07 fold. We were not able to categorize any specific single class of genes/proteins that contributed to the increase in core genome size over time, the whole list of unique genes for each of the three core genomes are presented in the Additional file 3: Table S1.

### The emergence of the modern *Neisseria gonorrhoeae*

**Fig. 5:**
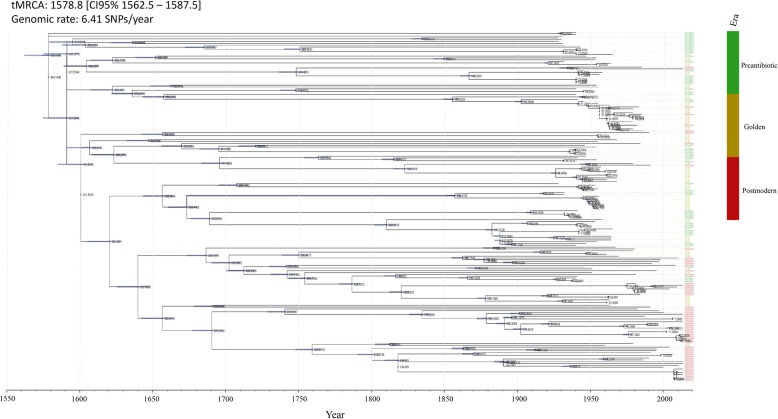
Maximum likelihood tree including internal node labels for viable *Neisseria gonorrhoeae* isolates (*n* = 191) from 1928 to 2013 dated using least-squares dating (LSD) software v0.3 and 18,779 SNPs, to estimate the rate and the dates of the input phylogeny given temporal constraints. The relationship in the phylogeny shows that the isolates from the postmodern era belonging to the multidrug-resistant clade alone have a most recent common ancestor relatively soon after the inferred emergence of the whole collection, within the late 17th – early eighteenth century

**Fig. 6:**
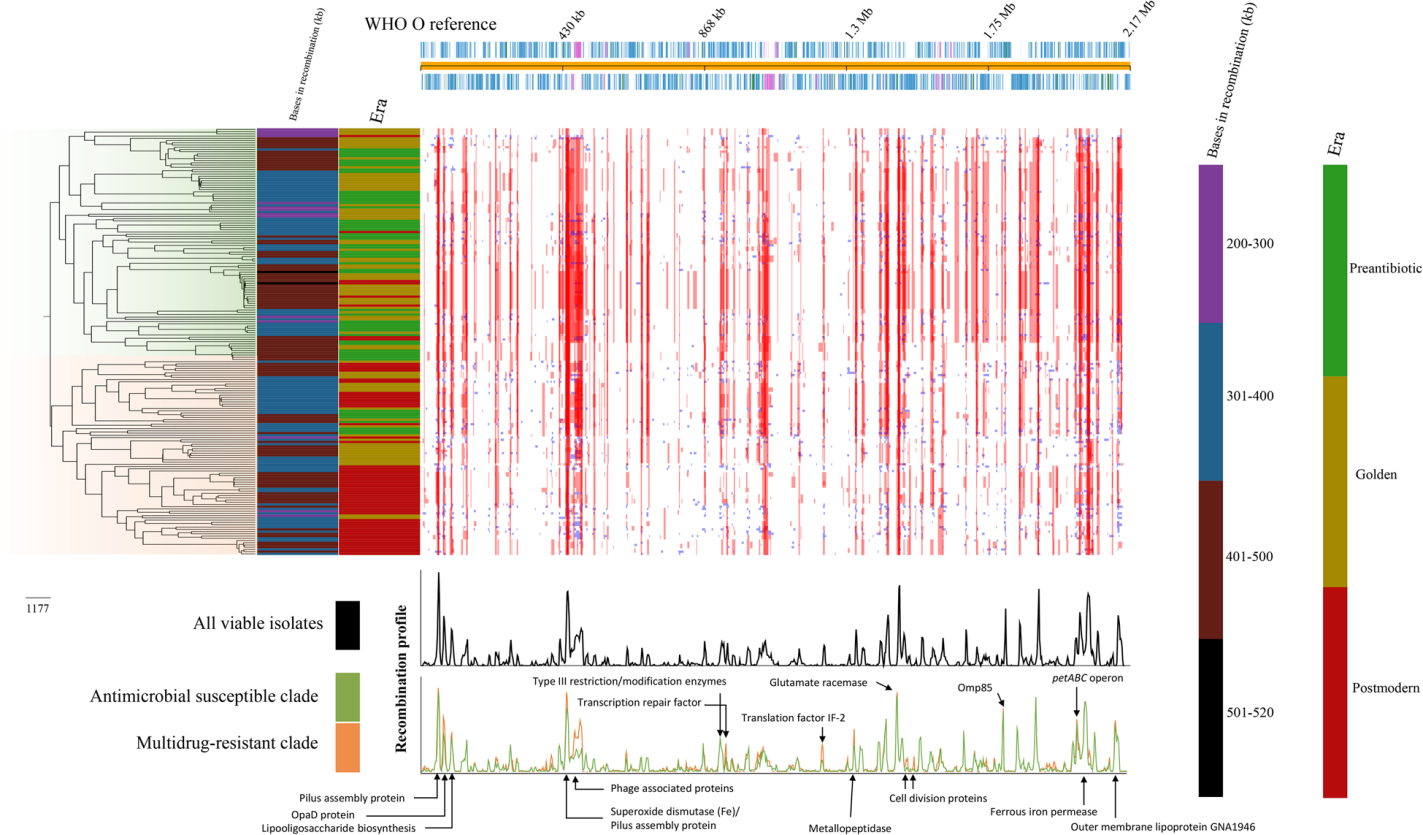
Phylogeny and recombination landscape for viable *Neisseria gonorrhoeae* isolates (*n* = 191). The phylogeny (left), with associated level of recombination and era of isolation, is displayed alongside the linearized chromosome of WHO O reference strain (top). Line graph (bottom) shows the recombination profile of all isolates as well as separately for the antimicrobial susceptible and multidrug-resistant clade. The number of recombination nucleotide sites ranged from 213,780–539,280 sites, with a mean number of 388,811 sites and median of 393,987 sites

In a temporal analysis including all viable (*n* = 191) isolates, we estimated the substitution rate to 4.54E-6 substitutions/site/year, which translates to an accumulation of ~ 6.4 SNPs per year. The analysis was based on 1,411,028 nucleotide sites and 18,779 SNPs after homologous recombination, repeat regions, and phage sequences were masked and removed. The calibrated phylogeny (Fig. 5) dated the most recent common ancestor (tMRCA) of the analyzed dataset in the late sixteenth century (1579; CI95% 1562–1588). The temporal analysis for the antimicrobial susceptible clade (green, Fig. 3) (*n* = 107) estimated the substitution rate of ~ 3.10E-6 (6.8 SNPs/year) and the tMRCA to 1142 (CI95% 976–1280). For the AMR clade (red, Fig. 3) (*n* = 84), a substitution rate of ~ 8.31E-6 (18.3 SNPs/year) and a tMRCA at 1707 (CI95% 1703–1738) was estimated. This analysis suggested that the modern gonococcus is not as old as previously hypothesized and supports that strains from the postmodern era have evolved from strains belonging to the preantibiotic era and mainly form a separate clade in the tree. Furthermore, the isolates seemed to become increasingly clonal over time, which correlates with a more conserved genome. Interestingly, the level of recombination in isolates from the different eras did not substantially differ (Fig. 6), but there were differences in the recombination profile between the antimicrobial susceptible and the AMR clade. The regions differently affected by recombination included, for example, pilus assembly proteins, outer membrane proteins such as OpaD and GNA1946, type III restriction/modification enzymes, and hypothetical/uncharacterized proteins (Fig. 6). Studies further examining these regions in a large number of isolates belonging to the different clades would be valuable.

## Discussion

We describe the evolution of *N. gonorrhoeae* and its AMR within the Danish gonococcal population by examining the genomes of isolates cultured over nine decades (1928 to 2013), which represents the oldest collection of gonococcal isolates globally to our knowledge. The high access to antimicrobials in the Danish human population has been similar to most other Western countries over the past century. The *N. gonorrhoeae* isolates were divided into eras reflecting the discovery or development of specific antimicrobials and our approach was to combine traditional phenotypic methods with WGS, unifying the data and knowledge to get additional evidence of what might be important in the emergence, evolution, and spread of *N. gonorrhoeae* and its AMR*.*

Prior to the 1950s, sulfonamides and penicillins were the only available classes of antimicrobials for treatment of gonorrhoea in Denmark. These two antimicrobial classes were successfully used and, based on our data, no resistance to penicillin G was observed pre-1950s, while high MICs of sulfamethoxazole (64- > 256 mg/L) were observed in nearly 10% of isolates already in the 1940s. TMP-SMX was introduced in the early 1950s, however, most likely due to the overuse of sulfamethoxazole, the resistance to TMP-SMX was already present in the gonococcal population. The overuse in Denmark as in most countries caused strong concerns to be raised in the early 1960s about resistance to sulfonamides, while penicillin G was still described as “… a steady choice of drug against gonorrhoea” [28]. This statement is in concordance with our findings, which show < 5% resistance to penicillin G until the 1970s. However, the proportion of isolates with decreased susceptibility or resistance to macrolides such as erythromycin in the preantibiotic era was surprisingly high. We show that the first isolate with erythromycin resistance and exactly at the breakpoint for azithromycin resistance (ECOFF > 1 mg/L) was cultured in 1930, however, no known tested macrolide resistance mutations were found. Macrolides were not discovered at this time, however, macrolides, such as erythromycin, are a class of natural products and an environmental exposure selecting for increased macrolide MICs might explain these findings. During the golden era, when many of the antimicrobial classes we use today were discovered, resistance to sulfamethoxazole, penicillin G, erythromycin, and tetracycline became prevalent (up to 20%). In 1967, ampicillin replaced penicillin G for treatment of gonorrhoea in Denmark and in 1972 the Danish treatment guidelines changed to recommend first-line treatment with pivampicillin 1.4 g combined with probenecid 1 g, but also testing for susceptibility to penicillin G and treating penicillin G-resistant infections with spectinomycin 2 g, alternatively ESCs or ciprofloxacin. This first-line treatment was likely effective until the emergence and spread of both plasmid- and chromosomally-mediated resistance to penicillins during the late-1980s in the postmodern era. The majority of the known AMR determinants emerged during the postmodern era at an alarming rate resulting in an increase in resistance to many antimicrobials (Figs. 1 and 2, Table 1). Among the 14 investigated isolates from the 1990s, > 5% of isolates were resistant to penicillins (*n* = 5), TMP-SMX (*n* = 7), chloramphenicol (*n* = 6), tetracycline (*n* = 9), ciprofloxacin (*n* = 2), erythromycin (*n* = 8), cefuroxime (*n* = 1), and cefotaxime (*n* = 1). This is also reflected by the associated AMR determinants detected in the isolates and the change in treatment guidelines in Denmark to ceftriaxone 250 mg alternatively ciprofloxacin or spectinomycin to treat penicillin G-resistant gonorrhoea in the early-1990s. By the end of the 1990s, spectinomycin was excluded from the recommended treatment and only ceftriaxone 250 mg or ciprofloxacin 500 mg was recommended (Fig. 1). As in many countries, ceftriaxone was subsequently the only drug left for empiric monotherapy in the twenty-first century and during 2000–2013 the emergence of mosaic *penA* alleles, *gyrA* mutations and plasmid-mediated resistance were very common. Since 2015, dual antimicrobial therapy with ceftriaxone 500 mg plus azithromycin 2 g is recommended for treatment of gonorrhoea in Denmark (Fig. 1).

Surprisingly, the *penB* G120D AMR determinant, which has been described as one of the AMR determinants involved in penicillin and ESC resistance [4], was found in an isolate from 1928, i.e. from the preantibiotic era. This suggests that *penB* is not only selected by antimicrobial pressure but can also be selected due to its importance for many physiological functions and possibly fitness during infection. However, the MIC of penicillin G in this isolate was only 0.032 mg/L and it is not clear if the *penB* variant affected the MICs of any of the investigated antimicrobials. It has been earlier shown that the *penB* AMR determinant can require an overexpressed MtrCDE efflux pump to significantly increase the phenotypic MIC [4]. In contrast, it seems that the loss of function mutations in *mtrR,* a protein that also regulates many other functions [29, 30], is not found in strains before the wide use of antibiotics and, in our strain material, these mutations did not appear to have been selected by host-derived antibacterial agents such as fatty acids and bile salts, which has been previously suggested [29, 30]. Notably, the clinical isolates from 1928 might be the closest we can come to true gonococcal wild-type strains and opens up possibilities for further evolutionary studies. We also show that isolates cultured as early as 1940 contained amino acid alterations in the active site for ciprofloxacin (GyrA S91T), despite maintained susceptibility to ciprofloxacin. This mutation has consequences when predicting susceptibility to ciprofloxacin by detecting *gyrA* wild-type sequence with PCR-based tests, i.e. the lack of GyrA S91 wild-type sequence would result in a false report of ciprofloxacin resistance. The GGI was present in isolates across all nine decades (Fig. 2), and we did not identify any association between GGI and AMR or any particular AMR phenotype in contrast to a previous study [16]. Finally, we identified the human L1 element sequences [25] in 13 isolates, spanning back to 1946. Further studies might reveal how the human L1 element sequences got incorporated in the gonococcal genome and if the L1 element might be beneficial for *N. gonorrhoeae*.

Our phylogenomic analysis (Figs. 2 and 3) clearly showed how the MDR isolates from the postmodern era grouped in the same clade in the tree, while isolates from the preantibiotic and golden era were more diverse and formed no distinct clade(s). These MDR isolates from the postmodern era have evolved throughout the century, driven by the antimicrobial use/misuse, to form this MDR clade and some strains in this clade have now also developed or acquired resistance to ESCs. This MDR clade had a slightly different recombination profile and higher genomic mutation rate and accordingly is estimated to be more prone to adapt, e.g. from selective pressure of antimicrobial exposure. Additional genomic studies with recent global collection(s) of *N. gonorrhoeae* isolates would be valuable to define this MDR clade in detail, for early detection of strains with a predisposition to develop and maintain MDR and initiation of public health interventions.

The main difference between the isolates belonging to the different eras is the exposure to antimicrobials and most likely prolonged gonococcal infections before the postmodern era. A substantial number of AMR determinants and compensatory alterations in the genome remain unknown and further studies could provide an enhanced understanding of these AMR determinants as well as the diversity of *N. gonorrhoeae* [31]. The lack of known AMR determinants to explain some of the AMR detected further stresses the importance of maintaining phenotypic AMR testing. Thus, it appears that antimicrobial exposure and subsequently emerged resistance have been the significant overall drivers in the evolution, including species adaptation and diversification, of *N. gonorrhoeae*. This finding is further strengthened by the notion that the genome of the gonococcus became increasingly conserved, with an increasing core genome and a decrease of nucleotide diversity over time. The core genome increased from 1016 core genes to 1542 core genes (Fig. 4) and the nucleotide diversity decreased from 0.002644 (15,885 polymorphisms) to 0.002466 (12,862 polymorphisms) from the preantibiotic to the postmodern era, respectively. The total core genome for all viable isolates (1242 genes) over the nine decades can be described as the essential core genome for the species (Additional file 2: Figure S2), i.e., genes that are conserved over time despite antimicrobial use/misuse. Due to the stability of these genes across the species and over time, several of these could be promising gonococcal vaccine candidate antigens. In a comparison between the antibiotic eras, we found that many core genes are unique for isolates in preantibiotic, golden, and postmodern era (Additional file 3: Table S1). This further indicates that *N. gonorrhoeae* is evolving into a less diverse species, likely with enhanced fitness, but this enhanced fitness might be challenging to elucidate [32]. Studies regarding the biological fitness of isolates from preantibiotic and postmodern era can elucidate if and why certain clones are more fit and in general successful in their survival and transmission.

## Conclusions

WGS of gonococci from 1928 to 2013 showed that no AMR determinants, except *penB*, were in detectable frequency before the introduction of gonorrhoea therapeutic antimicrobials. The currently circulating modern gonococcus is substantially younger (tMRCA: 1579; CI95% 1562–1588) than previously hypothesized, which was also indicated in one of our most recent papers [31], and has since its emergence, evolved into a more clonal species driven by the use/misuse of antimicrobial treatment of gonorrhoea, but likely also of additional infections. Furthermore, the gonorrhoea treatment recommended and used appeared to select for different gonococcal clades and most AMR develops in strains belonging to one MDR clade. This clade should be further investigated for early detection of strains with predispositions to develop and maintain MDR and initiation of public health interventions.

## Supplementary information

**Additional file 1.** Supplementary methods including DNA extraction, antimicrobial resistance breakpoints and bioinformatic analysis.

**Additional file 2: Figure S1.** Number of whole-genome sequenced *Neisseria gonorrhoeae* specimens per decade. **Figure S2.** Pan-genome of all *Neisseria gonorrhoeae* specimens (*n*=231) showing the core genome of 1242 genes that were conserved over nine decades. Core= ≥99–100% of isolates share genes. Soft core = ≥95- < 99% of isolates share genes. Shell = ≥15- < 95% of isolates share genes. Cloud = 0- < 15% of isolates share genes.

**Additional file 3: Table S1.** Unique genes present in the core genome of the three eras. **Table S2.** European Nucleotide Archive (ENA) accession number for *Neisseria gonorrhoeae* isolates included in this study.

## Data Availability

All genomic data have been deposited in the European Nucleotide Archive (ENA) under project number PRJEB4024. Accession numbers for all examined isolates are provided in Supplementary Table 2.

## Abbreviations

AMR: Antimicrobial resistance
ECOFF: Epidemiologic cutoff value
ESCs: Extended-spectrum cephalosporins
EUCAST: European Committee on Antimicrobial Susceptibility Testing
MALDI-TOF-MS: Matrix-assisted laser desorption/ionization time-of-flight mass spectrometry
MDR: Multidrug resistance
MIC: Minimum inhibitory concentration
MLST: Multi-locus sequence typing
NG-MAST: *Neisseria gonorrhoeae* multi-antigen sequence typing
NG-STAR: *Neisseria gonorrhoeae* sequence typing for antimicrobial resistance
SNP: Single-nucleotide polymorphism
SSI: Statens serum institut
ST: Sequence type
TMP-SMX: Trimethoprim/sulfamethoxazole
tMRCA: The most recent common ancestor
WGS: whole-genome sequencing
WHO: World Health Organization
