# Supplementary material for: Genomic evolution of Neisseria gonorrhoeae since the preantibiotic era (1928–2013): antimicrobial use/misuse selects for resistance and drives evolution
Source: BMC Genomics. 2020 Feb 3;21:116. doi: 10.1186/s12864-020-6511-6 (PMC6998845; doi:10.1186/s12864-020-6511-6)
Supplement: Supplementary file 1 — Additional file 1. Supplementary methods including DNA extraction, antimicrobial resistance breakpoints and bioinformatic analysis. [file 12864_2020_6511_MOESM1_ESM.docx]

**Additional file 1: Supplementary Methods**

**DNA extraction**

All isolates and suspensions from freeze dried non-viable isolates were extracted using Wizard Genomic DNA Purification Kit (Promega Corporation, Madison, WI, USA), according to the manufacturer’s instructions with minor modifications. Briefly, samples were RNase-treated (4 µl of RNase) for 60 min, and 450 µl of RNA/protein-free DNA was further treated with isopropanol and ethanol. Finally, the DNA pellets were dehydrated for at least 2 h before adding DNA Rehydration Solution.

**Antimicrobial susceptibility and resistance breakpoints**

Antimicrobial susceptibility data were interpreted using EUCAST clinical breakpoints v9.0 (www.eucast.org/clinical_breakpoints). For azithromycin and erythromycin, no clinical breakpoints exist and the EUCAST epidemiological cut-off value (ECOFF) of 1 mg/L for azithromycin (www.eucast.org/clinical_breakpoints) was used to indicate resistance to azithromycin and erythromycin. Breakpoints for chloramphenicol, kanamycin, and trimethoprim-sulfamethoxazole were according to WHO guidelines [1].

The MICs were divided into upper (U), medium (M), and lower (L) range when clinical breakpoints where not available, i.e. for sulfamethoxazole (L: MIC<64, M: 64-256, U: >256 mg/L), ampicillin (L: MIC<2, M: 2-32, U: >32 mg/L), rifampin (L: MIC<2, M: 2-32, U: >32 mg/L), and ertapenem (L: MIC<0.064, M: 0.064-0.125, U: >0.125 mg/L).

**Bioinformatic analysis**

***Quality controls***

All sequencing reads were initially examined using standard Kraken database based on the Refseq bacterial, archeal, and viral genomes in February 2016, for species confirmation and identification of contamination [2]. Furthermore, analysis of heterozygosity was performed to exclude mixed cultures. Fifteen and 26 samples were excluded due to poor quality and contaminated sequences, respectively, leaving 231 samples (191 viable and 40 non-viable isolates) for downstream analysis. All sequences generated in this study have been submitted to European Nucleotide Archive (ENA) under the accession number PRJEB4024.

***Resistome and molecular epidemiological characterisation***

Paired-end reads from all isolates (n=231) were *de novo* assembled using SPAdes (v.2.4.0) with the --careful pipeline option [3] and a customized CLC Genomics Workbench 9.5.3 (https://www.qiagenbioinformatics.com/) with automatic graph parameters as part of two different workflows. The *de novo* assemblies generated by SPAdes (v.2.4.0) [3] were used for *in silico* determination of *N. gonorrhoeae* multi-antigen sequence typing (NG-MAST) using NGMASTER [4]. Screening for AMR markers including *mtrD* mosaics, mutations in *rplD and rplV* [5]*,* and acquisition of *ereA*/*ereB*, *erm*, and *mefA* genes was performed using an *in silico* PCR method (https://github.com/simonrharris/in_silico_pcr). Customized CLC Genomics Workbench 9.5.3 workflow and databases were used to extract *penA, ponA, gyrA, parC, rpsJ, mtrR, porB1b/porB1a* (corresponds to the serogroups WI or WII/III), *penB, pilQ, folP, rpoB, bla*_TEM_, *tetM,* the human L1 element, and alleles included in the multi-locus sequence typing (MLST) scheme (n=7) [6] and the *N. gonorrhoeae* Sequence Typing for Antimicrobial Resistance (NG-STAR) scheme (n=7) [7]. MLST eBURST profiles were obtained using eBURST v3 [6]. The allele frequencies of 23S rRNA and 16S rRNA gene resistance mutations (*Escherichia coli* numbering was used) were estimated using the integrated mapping and quality-based variant detection (Neighborhood Quality Standard algorithm) within the customized CLC Genomics Workbench 9.5.3 workflow with default parameters. Gonococcal genetic islands (GGIs) were identified using the PubMLST website (https://pubmlst.org/neisseria/).

***Phylogenomics and dating analysis***

Illumina reads from each isolate were used to find the best reference genome for this dataset among the 2016 WHO *N. gonorrhoeae* reference genomes (n=14) [8] and FA1090, by identifying the best matches using k-mer spectras using CLC Genomics Workbench with the k-mer length of 16 and only index k-mers with prefix ATGAC. Subsequently, reads were mapped to the best matched reference genome (WHO O) using BWA-MEM with minimum seed length of 19, penalty for mismatch 3 and the -M option to mark shorter splits hits as secondary [9], single-nucleotide polymorphisms (SNPs) were called using SAMtools (v1.3) mpileup with the -L 1000 -d 1000 -m options and BCFtools (v1.2) using call -A -M -v -S parameters, and a maximum-likelihood phylogenetic tree based on vertically inherited SNPs was obtained using RAxML (v8.2.8) using the gamma distribution model. Recombinant regions were identified and removed from the alignment using Gubbins (v1.4.10) with default parameters, 10 iterations and starting tree option given the output of RAxML [10]. Repeats and phage-associated regions were also masked from the alignment, which was further improved with Gblocks (v0.91b) with default parameters [11]. The temporal signal was evaluated using TempEst [12] by examining the root-to-tip genetic distance against the year of isolation for all viable isolates, as well as for the antimicrobial susceptible clade and the multidrug-resistant clade. The temporal signal for the three separated datasets was visually inspected but also informed by the r^2^ value that was 0.656, 0.245, and 0.480 for all viable isolates, the antimicrobial susceptible clade and the multidrug-resistant clade, respectively. Finally, molecular dating of ancestral events was performed using the least-squares dating (LSD) software v0.3 with -c -v 1 –s 18779 -t 1e-10 -f 1000 parameters –r as [13] after testing for temporal signal using a clustered permutation approach [14].

Prokka (v1.11) [15] was used to annotate the SPAdes (v2.4.0) [3] assembled genomes with default parameters with the –genus Neisseria option and Roary (v3.6.6) [16] was used to define the core genomes with -e -n options, universally for all isolates as well as separately for each era. The core genome (1091884 bp) of all isolates was divided into the three separate eras to calculate the nucleotide diversity for each era separately using Tajima’s Neutrality Test [17] conducted on MEGA X software [18].

Phylogenetic trees were midpoint rooted using FigTree (v1.4.4) and visualized with phandango (v1.3) [19].

**Supplementary references**

1. Gonorrhoea. In: Laboratory diagnosis of sexually transmitted diseases. World Health Organization (WHO). https://apps.who.int/iris/bitstream/handle/10665/41847/9241545011_eng.pdf. Accessed 22 September 2020.
2. Wood DE, Salzberg SL. Kraken: ultrafast metagenomic sequence classification using exact alignments. Genome Biol. 2014;15:R46.
3. Bankevich A, Nurk S, Antipov D, Gurevich AA, Dvorkin M, Kulikov AS, et al. SPAdes: a new genome assembly algorithm and its applications to single-cell sequencing. J Comput Biol. 2012;19:455-77.
4. Kwong JC, Gonçalves da Silva A, Dyet K, Williamson DA, Stinear TP, Howden BP, et al. NGMASTER: in silico multi-antigen sequence typing for *Neisseria gonorrhoeae*. Microb Genom 2016; doi:10.1099/mgen.0.000076.
5. Grad YH, Harris SR, Kirkcaldy RD, Green AG, Marks DS, Bentley SD, et al. Genomic epidemiology of gonococcal resistance to extended-spectrum cephalosporins, macrolides, and fluoroquinolones in the United States, 2000-2013. J Infect Dis. 2016;214:1579-87.
6. Feil EJ, Li BC, Aanensen DM, Hanage WP, Spratt BG. eBURST: inferring patterns of evolutionary descent among clusters of related bacterial genotypes from multilocus sequence typing data. J Bacteriol. 2004;186:1518-30.
7. Demczuk W, Sidhu S, Unemo M, Whiley DM, Allen VG, Dillon JR, et al. *Neisseria gonorrhoeae* Sequence Typing for Antimicrobial Resistance (NG-STAR): A novel antimicrobial resistance multilocus typing scheme for tracking the global dissemination of *N. gonorrhoeae* strains. J Clin Microbiol. 2017;55:1454-68.
8. Unemo M, Golparian D, Sánchez-Busó L, Grad Y, Jacobsson S, Ohnishi M, et al. The novel 2016 WHO *Neisseria gonorrhoeae* reference strains for global quality assurance of laboratory investigations: phenotypic, genetic and reference genome characterization. J Antimicrob Chemother. 2016;71:3096-108.
9. Li H. Aligning sequence reads, clone sequences and assembly contigs with BWA-MEM. arXiv:13033997v2 [q-bioGN]. 2013.
10. Croucher NJ, Page AJ, Connor TR, Delaney AJ, Keane JA, Bentley SD, et al. Rapid phylogenetic analysis of large samples of recombinant bacterial whole genome sequences using Gubbins. Nucleic Acids Res. 2015;43:e15.
11. Talavera G, Castresana J. Improvement of phylogenies after removing divergent and ambiguously aligned blocks from protein sequence alignments. Syst Biol. 2007;56:564-77.
12. Rambaut A, Lam TT, Max Carvalho L, Pybus OG. Exploring the temporal structure of heterochronous sequences using TempEst (formerly Path-O-Gen). Virus Evolution. 2016; doi: 10.1093/ve/vew007.
13. To T, Jung M, Lycett S, Gascuel O. Fast dating using least-squares criteria and algorithms. Syst Biol. 2016;1:82-97.
14. Murray GG, Wang F, Harrison EM, Paterson GK, Mather AE, Harris SR, et al. The effect of genetic structure on molecular dating and tests for temporal signal. Methods Ecol Evol. 2016;7: 80-9.
15. Seemann T. Prokka: rapid prokaryotic genome annotation. Bioinformatics. 2014;30:2068-9.
16. Page AJ, Cummins CA, Hunt M, Wong VK, Reuter S, Holden MT, et al. Roary: rapid large-scale prokaryote pan genome analysis. Bioinformatics. 2015;31:3691-3.
17. Tajima F. Statistical method for testing the neutral mutation hypothesis by DNA polymorphism. Genetics. 1989:123:585-95.
18. Kumar S, Stecher G, Li M, Knyaz C, Tamura K. MEGA X: Molecular Evolutionary Genetics Analysis across computing platforms. Mol Biol Evol. 2018:35:1547-9.
19. Hadfield J, Croucher NJ, Goater RJ, Abudahab K, Aanensen DM, Harris SR. Phandango: an interactive viewer for bacterial population genomics. Bioinformatics. 2017; doi:10.1093/bioinformatics/btx610.
