# Supplementary material for: Genomic evolution of Neisseria gonorrhoeae since the preantibiotic era (1928–2013): antimicrobial use/misuse selects for resistance and drives evolution
Source: BMC Genomics. 2020 Feb 3;21:116. doi: 10.1186/s12864-020-6511-6 (PMC6998845; doi:10.1186/s12864-020-6511-6)
Supplement: Supplementary file 2 — Additional file 2: Figure S1. Number of whole-genome sequenced Neisseria gonorrhoeae specimens per decade. Figure S2. Pan-genome of all Neisseria gonorrhoeae specimens (n=231) showing the core genome of 1242 genes that were conserved over nine decades. Core= ≥99–100% of isolates share genes. Soft core = ≥95- < 99% of isolates share genes. Shell = ≥15- < 95% of isolates share genes. Cloud = 0- < 15% of isolates share genes. [file 12864_2020_6511_MOESM2_ESM.docx]

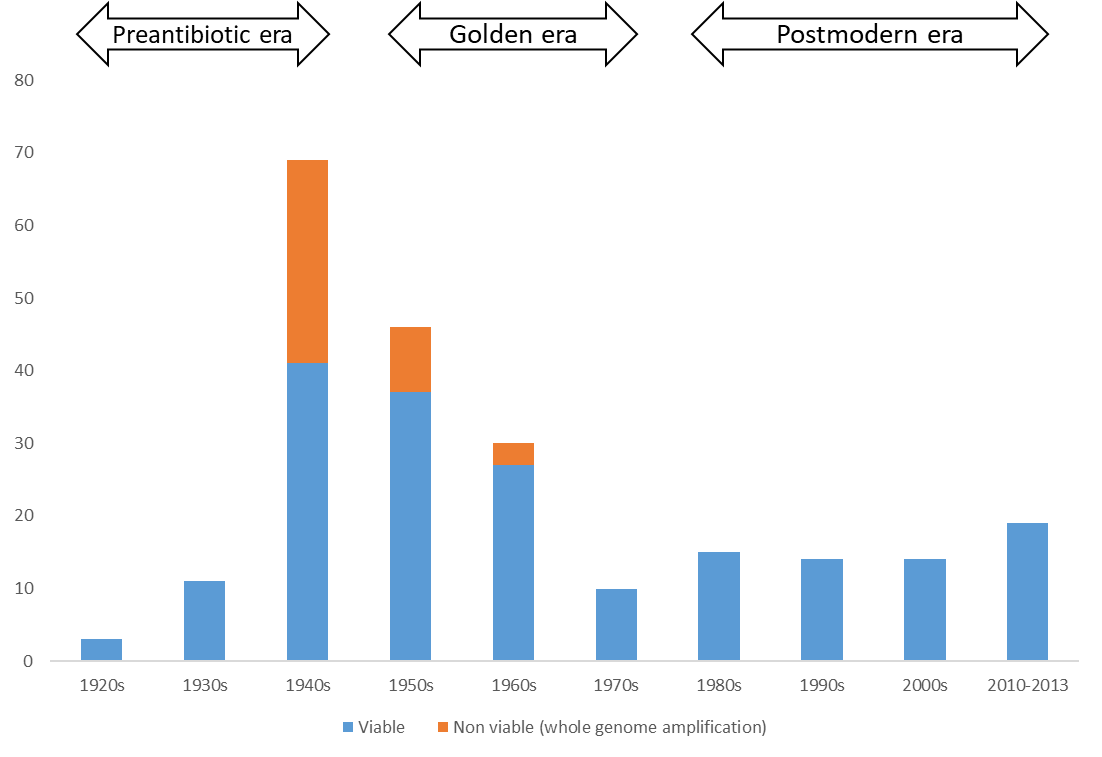


**Figure S1**. Number of whole genome sequenced *Neisseria gonorrhoeae* specimens per decade.


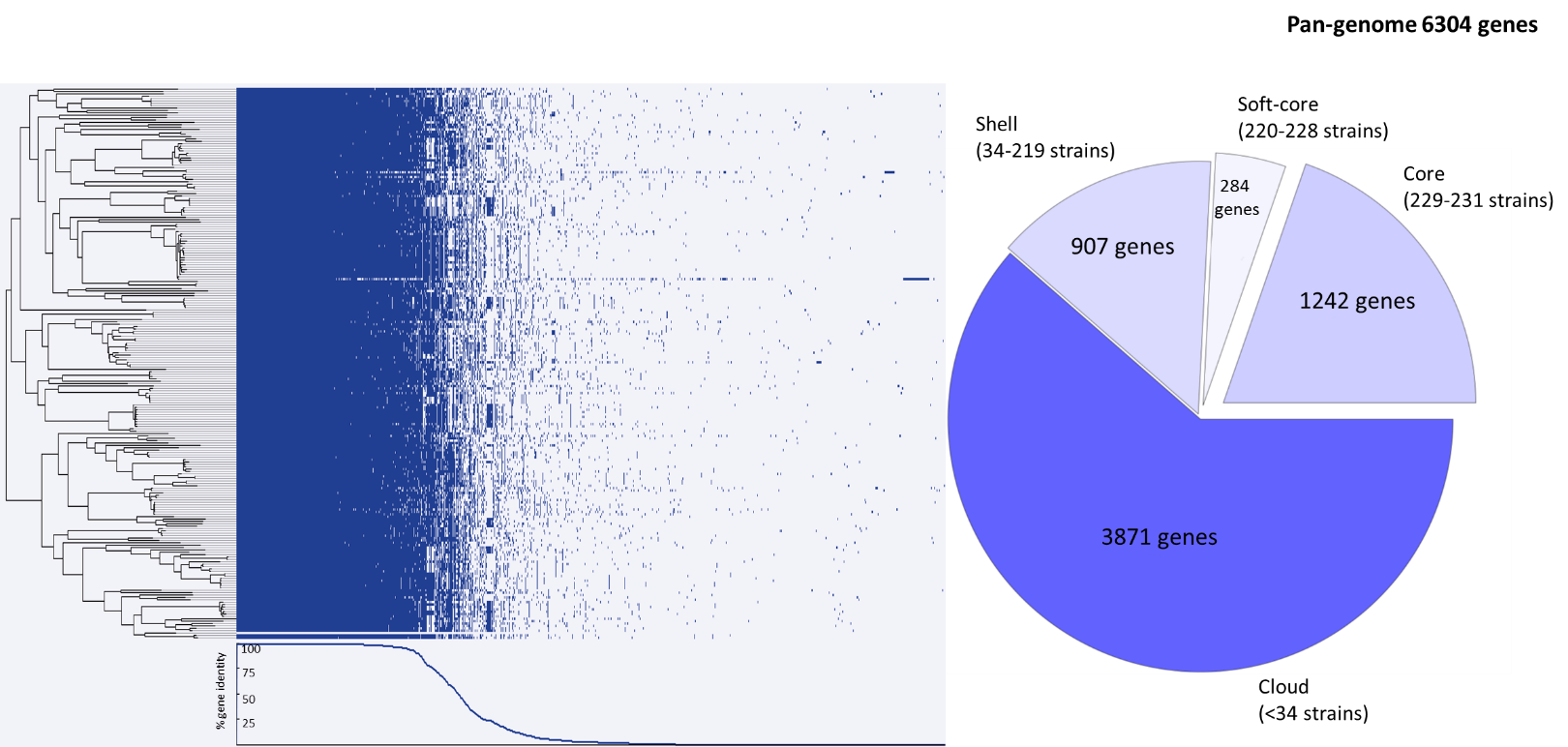


**Figure S2**. Pan-genome of all *Neisseria gonorrhoeae* specimens (n=231) showing the core genome of 1242 genes that were conserved over nine decades. Core= ≥99-100% of isolates share genes. Soft-core= ≥95-<99% of isolates share genes. Shell= ≥15-<95% of isolates share genes. Cloud= ≥0-<15% of isolates share genes.
