# Supplementary material for: Genomic evolution of Neisseria gonorrhoeae since the preantibiotic era (1928–2013): antimicrobial use/misuse selects for resistance and drives evolution
Source: BMC Genomics. 2020 Feb 3;21:116. doi: 10.1186/s12864-020-6511-6 (PMC6998845; doi:10.1186/s12864-020-6511-6)
Supplement: Supplementary file 3 — Additional file 3: Table S1. Unique genes present in the core genome of the three eras. Table S2. European Nucleotide Archive (ENA) accession number for Neisseria gonorrhoeae isolates included in this study. [file 12864_2020_6511_MOESM3_ESM.docx]

**Table S1.** Unique genes present in the core genome of the three eras.

| Preantibiotic | | Golden antibiotic era | | Postmodern era | |
| --- | --- | --- | --- | --- | --- |
| Genes | Proteins | Genes | Proteins | Genes | Proteins |
| *carA* | carbamoyl phosphate synthase small subunit | *argJ* | bifunctional ornithine acetyltransferase/N-acetylglutamate synthase | *aat* | leucyl/phenylalanyl-tRNA--protein transferase |
| *clcA_2* | Chloride channel protein-related protein | *brkA* | Pertactin | *aldA* | aldehyde dehydrogenase A |
| *gap_1* | glyceraldehyde-3-phosphate dehydrogenase | *cls* | phopholipase D-family protein | *ampG* | AmpG protein |
| *glyA* | serine hydroxymethyltransferase | *cysJ_2* | putative sulfite reductase subunit alpha | *arfA* | Alternative ribosome-rescue factor A |
| group_1020 | polyphosphate kinase 2 | *cysW_1* | putative ABC transporter | *argS* | arginyl-tRNA synthetase |
| group_1194 | Lipoprotein | *dgkA* | diacylglycerol kinase | *artQ* | ABC transporter permease, amino acid |
| group_1222 | sodium-dependent transport protein | *fdm* | alcohol dehydrogenase | *atpG* | ATP synthase F0F1 subunit gamma |
| group_1337 | cbb3-type cytochrome c oxidase subunit II | *fhaC* | putative hemolysin activation protein HecB | *avtA* | valine--pyruvate transaminase |
| group_1354 | phage associated protein | *frmA* | alcohol dehydrogenase | *betP* | transmembrane transport protein BCCT family |
| group_1384 | Lipoprotein | *ftsY* | signal recognition particle protein | *bspRIM_1* | site-specific DNA-methyltransferase M.NgoVII |
| group_1454 | putative phage associated protein | *galM* | aldose 1-epimerase | *cah* | Cah |
| group_1500 | cadmium resistance protein | *gatC* | aspartyl/glutamyl-tRNA amidotransferase subunit C | *cfaD* | AraC family transcriptional regulator |
| group_1508 | putative transmembrane transport protein | group_1032 | putative RNA-binding protein | *cmpB* | ABC transporter permease |
| group_1522 | Ankyrin | group_1035 | stress-sensitive restriction system protein | *coaBC* | flavoprotein |
| group_1537 | putative phage associated protein | group_1055 | putative CsgG-like lipoprotein | *codB* | permease |
| group_1538 | membrane protein | group_1062 | NgoPII restriction and modification | *crcB* | camphor resistance protein CrcB |
| group_1539 | Transposase | group_1069 | competence protein | *ctaD* | cbb3-type cytochrome c oxidase subunit I |
| group_1541 | membrane protein | group_1079 | factor H binding protein | *cysA* | ABC transporter ATP-binding protein |
| group_1595 | Phage baseplate assembly protein V | group_1089 | hydrolase | *cysW* | putative ABC transporter |
| group_1649 | FMN oxidoreductase CC3083 | group_1100 | putative acetyltransferase | *dca* | division cluster competence-associated protein |
| group_1677 | membrane protein | group_1105 | site-specific recombinase | *def* | peptide deformylase |
| group_1679 | putative serotype-1-specific antigen | group_1117 | 6-pyruvoyl tetrahydrobiopterin synthase | *dipZ* | thiol:disulfide interchange protein |
| group_1723 | cadmium resistance protein | group_1141 | putative DNA polymerase III subunit | *dltB* | alginate O-acetylation - like protein |
| group_1766 | putative inner membrane protein | group_1142 | phage repressor, phage associated protein | *dnaG* | DNA primase |
| group_1767 | PhnO-like protein | group_1168 | putative endonuclease | *dsbB* | disulfide bond formation protein |
| group_1776 | Lipoprotein | group_1171 | integral membrane protein | *fadD_1* | long-chain-fatty-acid--CoA-ligase |
| group_1777 | membrane protein | group_1178 | acyl-CoA hydrolase | *fetB* | membrane transport solute-binding protein |
| group_1778 | sodium-dependent inner membrane transport protein | group_1181 | phage associated protein | *fmt* | methionyl-tRNA formyltransferase |
| group_1783 | inner membrane protein | group_1185 | outer membrane protein OmpU | *fnr* | Fnr family transcriptional regulator |
| group_1794 | Acetyltransferase | group_1193 | putative lipoprotein | *fokIM* | protein DamH |
| group_1795 | factor H binding protein | group_1195 | integral membrane protein | *ftsH* | cell division protein FtsH |
| group_1798 | secreted protein | group_1227 | phage associated protein | *galE* | UDP-glucose 4-epimerase |
| group_1799 | integral membrane protein | group_1235 | putative phage associated protein | *ggt_2* | gamma-glutamyltranspeptidase |
| group_1816 | phage associated protein | group_1282 | cadmium resistance protein | *glnE* | bifunctional glutamine-synthetase adenylyltransferase/deadenyltransferase |
| group_1826 | putative hydrolase | group_1320 | putative glycosyltransferase | *glnG* | two-component system transcriptional response regulator |
| group_1839 | Oxidoreductase, putative | group_1322 | putative thiosulfate sulfur transferase | *gntP* | gluconate permease |
| group_1840 | Transferase | group_1327 | Protein CrcB | group_1001 | putative RNA-binding protein |
| group_1848 | TonB | group_1331 | Putative hemolysin | group_1003 | type I restriction-modification system methyltransferase |
| group_1860 | putative metal-dependent phosphoesterase | group_1363 | uridylyltransferase | group_1016 | membrane protein |
| group_1861 | TonB-dependent receptor | group_1366 | Nickel-dependent hydrogenase, b-type cytochrome subunit | group_1018 | ABC transporter, permease protein, SbmA/BacA family |
| group_1879 | putative acetyltransferase | group_1371 | ABC transporter, permease protein, SbmA/BacA family | group_1025 | uridylyltransferase |
| group_1884 | membrane protein | group_1372 | putative hydrolase | group_1039 | Membrane protein |
| group_1891 | putative phage associated protein | group_1379 | putative DNA-binding protein | group_1042 | factor H binding protein |
| group_1899 | membrane protein | group_1380 | OpcA | group_1059 | outer membrane protein OmpU |
| group_1911 | secreted protein | group_1385 | integral membrane protein | group_1063 | RlpA-like protein |
| group_1913 | major outer membrane protein | group_1388 | putative Sm-like integral membrane protein | group_1067 | putative oxidoreductase |
| group_1925 | Lipoprotein | group_1393 | ABC transporter ATP-binding protein | group_1072 | Periplasmic protein |
| group_1951 | secreted protein | group_1404 | NTP binding protein | group_1074 | ABC transporter substrate-binding protein |
| group_1962 | membrane protein | group_1417 | lipoprotein | group_1090 | cupin family protein |
| group_1973 | amino-acid transporter sodium/alanine symporter | group_1422 | prolyl endopeptidase | group_1092 | PilM |
| group_1974 | integral membrane protein | group_1424 | putative outer membrane peptidase | group_1096 | TonB-dependent receptor |
| group_1977 | putative lipoprotein | group_1428 | Thiamin pyrophosphokinase-related protein | group_1104 | membrane protein |
| group_1986 | putative DNA polymerase III subunit | group_1432 | Thiamine biosynthesis protein ThiS | group_1124 | putative hemolysin activation protein HecB |
| group_1988 | putative ferredoxin | group_1451 | PilN | group_1126 | membrane protein |
| group_2026 | Haem utilisation protein | group_1452 | PilM | group_1129 | putative acetyltransferase |
| group_2127 | integral membrane protein | group_1461 | putative metalloprotease NMB0538 | group_1131 | Thiamine biosynthesis protein ThiS |
| group_2171 | carbonic anhydrase | group_1465 | membrane protein | group_1137 | Thiamin pyrophosphokinase-related protein |
| group_2174 | putative Sm-like integral membrane protein | group_1467 | protein PncA | group_1140 | membrane protein |
| group_2198 | periplasmic protein (possibly peptidoglycan-binding) | group_1468 | membrane protein | group_1146 | MafB alternative c terminus |
| group_2200 | putative outer membrane protein | group_1475 | DNA-binding protein | group_1149 | membrane protein |
| group_2211 | NTP binding protein | group_1476 | membrane protein | group_1151 | acetyltransferase |
| group_2226 | Lipoprotein | group_1478 | lipoprotein | group_1155 | periplasmic protein (possibly peptidoglycan-binding) |
| group_2229 | Helix-turn-helix family protein | group_1488 | membrane protein | group_1156 | putative inner membrane protein |
| group_2236 | putative uracil-DNA glycosylase | group_1493 | NADH dehydrogenase I subunit N | group_1158 | putative outer membrane protein |
| group_2241 | Putative hemolysin | group_1494 | Membrane protein | group_1159 | Lipoprotein |
| group_2246 | membrane protein | group_1496 | phage protein | group_1160 | hemerythrin HHE cation binding domain protein |
| group_2250 | putative rRNA methylase | group_1499 | phosphatidylserine decarboxylase | group_1163 | Cell wall-associated hydrolase |
| group_2260 | membrane protein | group_1510 | Cell wall-associated hydrolase | group_1164 | major outer membrane protein |
| group_2266 | putative RNA-binding protein | group_1511 | major outer membrane protein | group_1165 | putative adenylate cyclase |
| group_2271 | type I restriction-modification system methyltransferase | group_1512 | major outer membrane protein | group_1182 | lipoprotein |
| group_2275 | bacteriophage N4 receptor, outer membrane subunit | group_1513 | putative adenylate cyclase | group_1205 | Sulfite exporter TauE/SafE |
| group_2289 | phage associated protein | group_1516 | outer membrane protein OmpU | group_1209 | CreA protein |
| group_2305 | putative competence protein ComJ | group_1519 | phage associated protein | group_1210 | putative TonB-dependent receptor protein |
| group_2311 | phage associated protein | group_1523 | secreted protein | group_1217 | putative phage associated membrane protein |
| group_2330 | ProQ/FINO family | group_1528 | phopholipase | group_1219 | putative transposase |
| group_2340 | putative sugar transporter | group_1533 | ATP-binding protein | group_1224 | Membrane protein |
| group_2342 | putative metalloprotease NMB0538 | group_1543 | putative AcnD-accessory protein PrpF | group_1239 | phage repressor, phage associated protein |
| group_2349 | PilN | group_1550 | Periplasmic protein | group_1242 | putative ferredoxin |
| group_2357 | ABC transporter ATP-binding protein | group_1558 | aldose 1-epimerase | group_1246 | transglycosylase |
| group_2364 | putative oxidoreductase | group_1563 | Lipoprotein | group_1253 | Outer membrane protein (porin) |
| group_2366 | Thiamin pyrophosphokinase-related protein | group_1565 | putative outer membrane protein | group_1254 | putative N-acetyltransferase |
| group_2368 | Hydrolase | group_1569 | periplasmic protein (possibly peptidoglycan-binding) | group_1258 | outer membrane protein OmpU |
| group_2374 | Thiamine biosynthesis protein ThiS | group_1571 | Protein smg-like protein | group_1259 | membrane protein |
| group_2377 | membrane protein | group_1574 | Sulfite exporter TauE/SafE | group_1265 | maltose phosphorylase |
| group_2379 | Hemolysin, putative | group_1580 | surface exposed protein | group_1267 | phage associated protein |
| group_2383 | Periplasmic protein | group_1582 | putative phage associated membrane protein | group_1269 | putative lipoprotein |
| group_2385 | putative lysR-family transcriptional regulator | group_1583 | phage associated protein | group_1270 | Periplasmic protein |
| group_2386 | DNA-binding protein | group_1584 | phage associated protein | group_1271 | protein PacB |
| group_2387 | hemagglutinin/hemolysin-like protein | group_1587 | phage repressor protein, phage associated protein | group_1275 | secreted protein |
| group_2392 | putative tail fiber protein | group_1588 | phage associated protein | group_1285 | phage associated protein |
| group_2393 | Polyprotein | group_1590 | Periplasmic protein | group_1286 | Pertactin |
| group_2411 | Putative periplasmic protein | group_1638 | putative N-acetyltransferase | group_1289 | phage associated protein |
| group_2422 | putative adenylate cyclase | group_1642 | Copper ABC transporter, periplasmic copper-binding protein | group_1294 | putative integral membrane protein |
| group_2431 | Aminotransferase | group_1644 | outer membrane protein OmpU | group_1297 | phage associated protein |
| group_2481 | putative TonB-dependent receptor protein | group_1645 | putative lipoprotein | group_1350 | invertase related gene 7, phage associated protein |
| group_2483 | putative esterase | group_1652 | phage associated protein | group_155 | type IV pilus assembly protein PilX |
| group_2505 | ATP dependent DNA helicase | group_1656 | phage associated protein | group_225 | outer membrane protein |
| group_2510 | magnesium transporter | group_1688 | ankyrin | group_2462 | hemoglobin receptor |
| group_2511 | phage repressor, phage associated protein | group_1755 | sodium-dependent inner membrane transport protein | group_2464 | lipoprotein |
| group_2519 | putative N-acetyltransferase | group_1761 | RlpA-like protein | group_2469 | YCII domain protein |
| group_2522 | membrane protein | group_2988 | nitrous-oxide reductase precurser | group_2471 | lipoprotein |
| group_2524 | Copper ABC transporter, periplasmic copper-binding protein | group_2991 | putative methyltransferase | group_2473 | putative lysR-family transcriptional regulator |
| group_2528 | outer membrane protein OmpU | group_2994 | VacJ-like protein | group_2474 | integral membrane protein |
| group_2533 | putative lipoprotein | group_2998 | putative integral membrane protein | group_2484 | membrane protein |
| group_2547 | putative type I restriction-modification system DNA methylase | group_3000 | putative secreted protein | group_2488 | ACT domain-containing protein |
| group_2549 | putative polynucleotidyl transferase | group_3001 | Periplasmic protein | group_2491 | VacJ-like protein |
| group_2553 | putative integral membrane protein | group_3016 | Putative integral membrane protein | group_2492 | Periplasmic protein |
| group_2573 | putative lactoylglutathione-related protein | group_3017 | putative integral membrane protein | group_2498 | TPR repeat protein |
| group_2678 | PilM | group_3018 | cadmium resistance protein | group_2502 | Predicted permease, DMT superfamily |
| group_4595 | integral membrane protein | group_3022 | ProQ/FINO family | group_2507 | cadmium resistance protein |
| group_4597 | phage associated protein | group_3035 | Haem utilisation protein | group_2520 | type 4 pilus biogenesis protein |
| group_4601 | Membrane protein | group_3036 | putative zinc finger/helix-turn-helix protein, YgiT family | group_2526 | putative secreted protein |
| group_4602 | Putative integral membrane protein | group_3039 | Membrane protein | group_2532 | phage protein |
| group_4603 | Periplasmic protein | group_3052 | putative lysR-family transcriptional regulator | group_2534 | Putative integral membrane protein |
| group_4606 | putative DNA glycosylase | group_3054 | Membrane protein | group_2536 | Region found in RelA / SpoT proteins |
| group_4618 | putative DNA-binding protein | group_3056 | phage associated protein | group_2541 | Protein smg-like protein |
| group_4628 | Protein smg-like protein | group_3059 | putative rRNA methylase | group_2551 | tetraacyldisaccharide 4'-kinase |
| group_4629 | 4-oxalocrotonate tautomerase | group_306 | putative phage associated protein | group_2554 | Cb-type cytochrome c oxidase subunit IV |
| group_4631 | putative integral membrane protein | group_3066 | Periplasmic protein | group_2557 | putative inner membrane protein |
| group_4638 | Periplasmic protein | group_3071 | Glucose-6-phosphate 1-dehydrogenase (EC 1.1.1.49) | group_2561 | phage associated protein |
| group_4642 | type 4 pilus biogenesis protein | group_3078 | YCII domain protein | group_2563 | putative DNA-binding protein |
| group_4663 | tetraacyldisaccharide 4'-kinase | group_3089 | Hemolysin, putative | group_2572 | NrrF |
| group_4666 | Fe(2 )-trafficking protein | group_3094 | Membrane lipoprotein | group_2582 | Transposase and inactivated derivatives |
| group_4674 | putative acetyltransferase | group_3096 | Region found in RelA / SpoT proteins | group_2583 | 4-oxalocrotonate tautomerase |
| group_4676 | Periplasmic protein | group_3101 | phage associated protein | group_2584 | membrane protein |
| group_4678 | putative zinc finger/helix-turn-helix protein, YgiT family | group_3104 | 4-oxalocrotonate tautomerase | group_2589 | C-type cytochrome |
| group_4686 | Oxidoreductase | group_3112 | ATP synthase I | group_2593 | putative phage associated protein |
| group_4690 | putative cell division protein FtsN | group_3115 | methionine biosynthesis MetW | group_2595 | membrane protein |
| group_4692 | Protein CrcB | group_3117 | electron transfer flavoprotein-ubiquinone oxidoreductase | group_2601 | Glucose-6-phosphate 1-dehydrogenase (EC 1.1.1.49) |
| group_4695 | O-methyltransferase | group_3122 | putative uracil-DNA glycosylase | group_2604 | phage associated protein |
| group_4696 | Lipoprotein | group_3123 | putative HIT domain protein | group_2605 | dissimilatory nitrous oxide reduction protein, lipoprotein |
| group_4700 | Transglycosylase | group_3132 | membrane protein | group_2611 | integral membrane protein |
| group_4705 | putative phosphotransferase | group_3136 | aminotransferase | group_2616 | outer membrane protein P1 |
| group_4710 | Ton-B dependent receptor | group_3149 | Periplasmic protein | group_2624 | phage associated protein |
| group_4715 | Membrane protein | group_3150 | membrane protein | group_2627 | phage associated protein |
| group_4720 | cbb3-type cytochrome c oxidase subunit I | group_3158 | outer membrane protein, OMP85 family | group_2641 | integral membrane transport protein |
| group_4725 | Membrane protein | group_3167 | VacJ-like protein | group_2649 | membrane protein |
| group_4729 | Predicted periplasmic/secreted protein | group_3177 | HAD hydrolase | group_2660 | Periplasmic protein |
| group_4732 | Transporter | group_318 | putative lipoprotein | group_2664 | membrane protein |
| group_4733 | oxidoreductase, NAD(P)H-flavin | group_3180 | integral membrane protein | group_2668 | Fe(2 )-trafficking protein |
| group_4739 | putative phosphoribosyltransferase | group_3186 | genome-derived Neisseria antigen 1162 | group_2670 | cbb3-type cytochrome c oxidase subunit II |
| group_4740 | putative ATP-binding protein | group_3189 | oxidoreductase | group_2675 | Membrane protein |
| group_4750 | PilO | group_3191 | hemagglutinin/hemolysin-like protein | group_2685 | oxidoreductase |
| group_4764 | ZipA, C-terminal FtsZ-binding domain | group_3206 | putative phosphotransferase | group_2699 | integral membrane protein |
| group_4768 | genome-derived Neisseria antigen 1162 | group_3244 | peptidase | group_2701 | tetratricopeptide repeat protein |
| group_4775 | Lipoprotein | group_3249 | tetrapac protein | group_2709 | Periplasmic protein |
| group_4780 | protein PncA | group_3250 | hemoglobin receptor | group_2713 | AraC family transcriptional regulator |
| group_4781 | putative secreted protein | group_3251 | amino-acid transporter | group_2724 | putative glycosyltransferase |
| group_4796 | ABC transporter substrate-binding protein | group_3265 | ABC transporter ATP-binding protein | group_2742 | TonB |
| group_4799 | Periplasmic protein | group_3266 | MJ0042 family finger-like domain | group_2752 | integral membrane protein NnrS |
| group_4801 | nodulation efficiency NfeD family protein | group_3273 | transglycosylase | group_2755 | secreted protein |
| group_4813 | phosphatidylserine decarboxylase | group_3277 | putative phage associated protein | group_2758 | VacJ-like protein |
| group_4821 | membrane protein | group_3285 | membrane protein | group_2761 | Periplasmic protein |
| group_4826 | putative glycosyltransferase | group_3288 | ZipA, C-terminal FtsZ-binding domain | group_2765 | putative lipoprotein |
| group_4827 | ABC transporter ATP-binding protein | group_3295 | lipoprotein | group_2766 | ABC transporter ATP-binding protein |
| group_4836 | ATP synthase I | group_3301 | Periplasmic protein | group_2770 | ProQ/FINO family |
| group_4839 | cupin domain-containing protein | group_3307 | tetratricopeptide repeat protein | group_2787 | putative DNA glycosylase |
| group_4848 | Region found in RelA / SpoT proteins | group_3343 | phage associated protein | group_2789 | hemoglobin receptor |
| group_4849 | integral membrane protein | group_3348 | putative transmembrane transport protein | group_279 | recombinase B |
| group_4854 | Acetyltransferase | group_3351 | Predicted esterase of the alpha/beta hydrolase fold | group_2798 | electron transfer flavoprotein-ubiquinone oxidoreductase |
| group_4857 | integral membrane protein | group_3358 | lipoprotein | group_2802 | putative phage associated protein |
| group_4858 | MORN repeat protein | group_3359 | putative universal stress protein | group_2811 | O-methyltransferase |
| group_4861 | Cnp1 | group_3360 | putative hydrolase | group_2822 | putative metallopeptidase |
| group_4864 | ABC transporter, permease protein, SbmA/BacA family | group_3374 | putative transcriptional regulator FolI | group_2823 | putative serotype-1-specific antigen |
| group_4865 | Membrane protein | group_3378 | putative inner membrane protein | group_2825 | putative universal stress protein |
| group_4868 | VacJ-like protein | group_3386 | putative metallopeptidase | group_283 | phage associated protein |
| group_4870 | acyl-CoA hydrolase | group_3397 | Helix-turn-helix family protein | group_2832 | acyl-CoA hydrolase |
| group_4874 | tetratricopeptide repeat protein | group_3403 | putative phage associated protein | group_2833 | putative transcriptional regulator FolI |
| group_4879 | putative AsmA-like protein | group_3422 | integral membrane protein NnrS | group_2835 | GntR family transcriptional regulator |
| group_4891 | putative HIT domain protein | group_3423 | NgoI restriction endonuclease | group_2839 | Periplasmic protein |
| group_4893 | putative inner membrane protein | group_3424 | putative esterase | group_284 | sel1 repeat protein |
| group_4908 | membrane protein | group_3434 | esterase | group_2840 | site-specific recombinase |
| group_4910 | putative metallopeptidase | group_3436 | Cnp1 | group_2841 | phage associated protein |
| group_4933 | membrane protein | group_3438 | cbb3-type cytochrome c oxidase subunit I | group_2862 | Fic family protein |
| group_4935 | TonB-dependent receptor protein | group_3450 | Periplasmic protein | group_2866 | phage associated protein |
| group_4936 | Nickel-dependent hydrogenase, b-type cytochrome subunit | group_3454 | dissimilatory nitrous oxide reduction protein, lipoprotein | group_2867 | Periplasmic protein |
| group_4952 | GntR family transcriptional regulator | group_3460 | Inner membrane protein | group_2870 | roadblock/LC7 domain protein |
| group_4955 | outer membrane protein, OMP85 family | group_3468 | ABC transporter ATP-binding protein | group_2876 | putative uracil-DNA glycosylase |
| group_4956 | Predicted esterase of the alpha/beta hydrolase fold | group_3479 | sel1 repeat protein | group_2880 | putative AsmA-like protein |
| group_4959 | putative class-II glutamine amidotransferase | group_3483 | cupin domain-containing protein | group_2887 | putative acetyltransferase |
| group_4961 | tetrapac protein | group_3492 | putative serotype-1-specific antigen | group_2890 | putative phage associated protein |
| group_4963 | putative 4-hydroxybenzoate synthetase | group_3498 | integral membrane protein | group_2895 | transcriptional regulator |
| group_4970 | potassium/proton antiporter | group_3503 | secreted protein | group_2901 | sodium-dependent inner membrane transport protein |
| group_4976 | Glucose-6-phosphate 1-dehydrogenase (EC 1.1.1.49) | group_3514 | TonB-dependent receptor protein | group_2910 | NosY |
| group_4977 | Adhesin | group_3580 | Fic family protein | group_2913 | oxidoreductase |
| group_4985 | C-type cytochrome | group_3582 | putative ATP-binding protein | group_2927 | potassium/proton antiporter |
| group_4992 | site-specific recombinase | group_3595 | membrane protein | group_2930 | putative methyltransferase |
| group_5000 | 2-nitropropane dioxygenase | group_3597 | ABC transporter substrate-binding protein | group_2931 | Protein CrcB |
| group_5003 | putative phage associated protein | group_3618 | magnesium transporter | group_2938 | acyl-CoA hydrolase |
| group_5004 | phage associated protein | group_3619 | Predicted periplasmic/secreted protein | group_2946 | lipoprotein |
| group_5011 | deoxyribonucleotide triphosphate pyrophosphatase | group_3625 | 4-carboxymuconolactone decarboxylase | group_2948 | TonB-dependent receptor protein |
| group_5024 | integral membrane protein | group_3626 | putative ferredoxin | group_2956 | amino-acid transporter sodium/alanine symporter |
| group_5034 | Domain of Unknown Function (DUF1543) | group_3629 | Periplasmic protein | group_2958 | twitching motility/pilus retraction protein |
| group_5035 | integral membrane protein | group_3634 | Periplasmic protein | group_2959 | genome-derived Neisseria antigen 1162 |
| group_5044 | Copper ABC transporter, periplasmic copper-binding protein | group_3641 | integral membrane protein | group_2965 | ABC transporter ATP-binding protein |
| group_5045 | FAD-binding protein | group_3643 | putative phosphoribosyltransferase | group_2973 | Predicted esterase of the alpha/beta hydrolase fold |
| group_5046 | putative outer membrane protein | group_3663 | putative oxidoreductase | group_2975 | Predicted ATPase (AAA superfamily) |
| group_5048 | acyl-CoA hydrolase | group_3667 | ABC transporter substrate-binding protein | group_299 | phage associated protein |
| group_5051 | membrane protein | group_3671 | putative class-II glutamine amidotransferase | group_3006 | Predicted periplasmic/secreted protein |
| group_5052 | putative hydrolase | group_3674 | roadblock/LC7 domain protein | group_3008 | Putative integrase/recombinase phage associated protein |
| group_5054 | phage associated protein | group_3679 | putative lipoprotein | group_3021 | HAD hydrolase |
| group_5056 | ACT domain-containing protein | group_3680 | integral membrane protein | group_3028 | putative 4-hydroxybenzoate synthetase |
| group_5057 | ABC-type taurine transport system, periplasmic component | group_3696 | nodulation efficiency NfeD family protein | group_3032 | prolyl endopeptidase |
| group_5066 | CreA protein | group_3702 | putative polynucleotidyl transferase | group_3034 | membrane protein |
| group_5067 | Putative ring dioxygenase subunit beta | group_3708 | putative hydrolase | group_3055 | Periplasmic protein |
| group_5070 | TPR repeat protein | group_3719 | Predicted ATPase (AAA superfamily) | group_3082 | HolC protein |
| group_5071 | hemerythrin HHE cation binding domain protein | group_3730 | NosY | group_3093 | tetrapac protein |
| group_5076 | Protein rnfH | group_3732 | putative fimbrial assembly protein | group_3103 | magnesium transporter |
| group_5077 | phage associated protein | group_3736 | integral membrane protein | group_3107 | aminotransferase |
| group_5084 | putative inner membrane protein | group_3738 | putative inner membrane protein | group_3134 | MORN repeat protein |
| group_5091 | phage associated protein | group_3741 | membrane protein | group_3141 | cytochrome |
| group_5092 | YCII domain protein | group_3742 | secreted protein | group_3145 | methionine biosynthesis MetW |
| group_5095 | phage associated protein | group_3743 | putative lipoprotein | group_3148 | lipoprotein |
| group_5097 | putative endonuclease | group_3747 | phage associated protein | group_3151 | putative class-II glutamine amidotransferase |
| group_5110 | RlpA-like protein | group_3752 | phage associated protein | group_3155 | sel1 repeat protein |
| group_5112 | putative methyltransferase | group_3755 | oxidoreductase, NAD(P)H-flavin | group_3162 | outer membrane protein |
| group_5113 | outer membrane protein | group_3757 | putative outer membrane protein | group_3169 | outer membrane protein, OMP85 family |
| group_5114 | membrane protein | group_3759 | Lipoprotein | group_3173 | amino-acid transporter |
| group_5117 | putative ATPase | group_3761 | oxidoreductase | group_3182 | putative inner membrane protein |
| group_5118 | membrane protein | group_3767 | hemerythrin HHE cation binding domain protein | group_3188 | hemagglutinin/hemolysin-like protein |
| group_5126 | OpcA | group_3769 | Putative ring dioxygenase subunit beta | group_3190 | Periplasmic protein |
| group_5136 | putative type I restriction-modification system DNA methylase | group_3773 | ABC-type taurine transport system, periplasmic component | group_3207 | 2-nitropropane dioxygenase |
| group_5144 | Lipoprotein | group_3778 | acyl-CoA hydrolase | group_3209 | ABC transporter periplasmic binding protein, amino acid |
| group_5148 | putative rRNA methylase | group_3786 | TPR repeat protein | group_3212 | Membrane protein |
| group_5149 | putative phage associated protein | group_3791 | putative sugar transporter | group_3214 | Membrane protein |
| group_674 | sel1 repeat protein | group_3792 | ABC transporter periplasmic binding protein, amino acid | group_3215 | peptidase |
| group_806 | prolyl endopeptidase | group_3793 | HemY protein | group_322 | putative lipoprotein |
| *Hgd* | 3-hydroxyacid dehydrogenase | group_3798 | acetyltransferase | group_3221 | putative hydrolase |
| *hsdR_1* | putative type I restriction enzyme EcoR124II R protein | group_3801 | putative phage associated protein | group_3225 | putative phosphotransferase |
| *mutS* | DNA mismatch repair protein MutS | group_3807 | deoxyribonucleotide triphosphate pyrophosphatase | group_3236 | lipoprotein |
| *speA* | arginine decarboxylase | group_3813 | CreA protein | group_3252 | phopholipase |
|  |  | group_3815 | phage associated protein | group_3258 | transferase |
|  |  | group_3818 | Thioredoxin | group_3259 | 4-carboxymuconolactone decarboxylase |
|  |  | group_3821 | Protein rnfH | group_3268 | carboxypeptidase |
|  |  | group_3833 | phage associated protein | group_3276 | Peptidase propeptide and YPEB domain |
|  |  | group_3840 | putative secreted protein | group_3284 | membrane protein |
|  |  | group_3841 | carbonic anhydrase | group_3287 | nodulation efficiency NfeD family protein |
|  |  | group_3842 | cell division protein FtsL-related protein | group_3305 | lipoprotein |
|  |  | group_3844 | phage associated protein | group_3306 | Haem utilisation protein |
|  |  | group_3848 | integral membrane protein | group_331 | phage associated protein |
|  |  | group_3852 | putative cell division protein FtsN | group_3317 | transcriptional regulator |
|  |  | group_3856 | membrane protein | group_3318 | ZipA, C-terminal FtsZ-binding domain |
|  |  | group_3857 | phage associated protein | group_3321 | Membrane lipoprotein |
|  |  | group_3860 | MafB alternative c terminus | group_3323 | putative phage associated protein |
|  |  | group_3868 | tetraacyldisaccharide 4'-kinase | group_3324 | ABC-type taurine transport system, periplasmic component |
|  |  | group_3872 | invertase related gene 7, phage associated protein | group_3352 | oxidoreductase |
|  |  | group_3876 | membrane protein | group_3366 | membrane protein |
|  |  | group_3879 | membrane protein | group_3373 | putative ATPase |
|  |  | group_3887 | VanZ family protein | group_3377 | phosphatidylserine decarboxylase |
|  |  | group_3890 | putative metallopeptidase | group_3390 | putative fimbrial assembly protein |
|  |  | group_3891 | NrrF | group_3392 | HemY protein |
|  |  | group_3896 | putative ferredoxin | group_3393 | major outer membrane protein |
|  |  | group_3897 | cupin family protein | group_3396 | ATP-binding protein |
|  |  | group_3902 | putative rRNA methylase | group_340 | oxidoreductase, NAD(P)H-flavin |
|  |  | group_3920 | cytoplasmic membrane protein | group_3401 | phage associated protein |
|  |  | group_3922 | type 4 pilus biogenesis protein | group_3407 | putative cell division protein FtsN |
|  |  | group_3930 | outer membrane protein | group_3415 | FAD-binding protein |
|  |  | group_3931 | lipoprotein | group_3417 | phage integrase |
|  |  | group_3933 | putative methyltransferase | group_3426 | Thioredoxin |
|  |  | group_3934 | Oxidoreductase, putative | group_3428 | Copper ABC transporter, periplasmic copper-binding protein |
|  |  | group_435 | polyphosphate kinase 2 | group_3433 | putative outer membrane peptidase |
|  |  | group_541 | putative integral membrane protein | group_3443 | acetyltransferase |
|  |  | group_560 | sel1 repeat protein | group_3455 | DNA-binding protein |
|  |  | group_561 | repetitive large surface protein | group_3458 | NTP binding protein |
|  |  | group_565 | putative oxidoreductase | group_3462 | putative phage associated protein |
|  |  | group_584 | acetyltransferase | group_3463 | PilP protein |
|  |  | group_647 | putative competence protein ComJ | group_3464 | phage associated protein |
|  |  | group_649 | putative integral membrane protein | group_347 | Phosphoglycerol transferase and related proteins, alkaline phosphatase superfamily |
|  |  | group_659 | type III restriction/modification system enzyme | group_3473 | membrane protein |
|  |  | group_714 | putative integral membrane protein | group_3474 | putative secreted protein |
|  |  | group_726 | integral membrane protein | group_3478 | Putative ring dioxygenase subunit beta |
|  |  | group_793 | membrane protein | group_3480 | phage associated protein |
|  |  | group_797 | FMN oxidoreductase CC3083 | group_3491 | deoxyribonucleotide triphosphate pyrophosphatase |
|  |  | group_819 | putative phage associated protein | group_3495 | Lipoprotein |
|  |  | group_846 | putative transmembrane transport protein | group_3504 | outer membrane protein OmpU |
|  |  | group_860 | Bacterial capsule synthesis protein PGA_cap | group_3505 | phage associated protein |
|  |  | group_924 | putative sodium dependent ion transport protein | group_3506 | cell division protein FtsL-related protein |
|  |  | group_927 | phage associated protein | group_3509 | Periplasmic protein |
|  |  | group_928 | phage associated protein | group_3510 | integral membrane protein |
|  |  | group_936 | phage associated protein | group_3517 | putative phage associated protein |
|  |  | group_949 | putative sugar transporter | group_3521 | membrane protein |
|  |  | group_950 | membrane protein | group_3525 | outer membrane protein P1 |
|  |  | group_952 | PhnO-like protein | group_3529 | lipoprotein |
|  |  | group_976 | amino-acid transporter sodium/alanine symporter | group_3532 | Lipoprotein |
|  |  | *hdl IVa* | hydrolase | group_3535 | Oxidoreductase, putative |
|  |  | *hgpA_1* | hemoglobin receptor | group_3541 | putative methyltransferase |
|  |  | *kefC* | glutathione-regulated potassium-efflux system protein | group_3550 | phage associated protein |
|  |  | *maf* | Maf-like protein | group_3555 | VanZ family protein |
|  |  | *mleN* | integral membrane protein | group_3558 | putative hydrolase |
|  |  | *nqo3* | NADH dehydrogenase subunit G | group_3571 | putative rRNA methylase |
|  |  | *nucH* | nuclease | group_3576 | ATP synthase I |
|  |  | *nuoL* | NADH:ubiquinone dehydrogenase, L subunit | group_3577 | putative phage associated protein |
|  |  | *piiC_2* | outer membrane protein | group_3581 | phage associated protein |
|  |  | *pilT_1* | Twitching motility protein | group_3583 | ankyrin |
|  |  | *pilT_2* | twitching motility - like protein | group_369 | putative oxidoreductase |
|  |  | *pip* | proline iminopeptidase | group_374 | outer membrane preprotein PIIc |
|  |  | *porA_2* | porin | group_391 | repetitive large surface protein |
|  |  | *relA* | GTP pyrophosphokinase | group_436 | Fic/DOC family |
|  |  | *sbp_2* | putative sulfate-binding protein | group_452 | MafB-like protein |
|  |  | *sdcS* | NadC family protein | group_496 | cadmium resistance protein |
|  |  | *sinR* | transcriptional regulator | group_497 | polyphosphate kinase 2 |
|  |  | *sppA* | protease | group_502 | 6-pyruvoyl tetrahydrobiopterin synthase |
|  |  | *ssuB* | ABC transporter ATP-binding protein | group_518 | phage associated protein |
|  |  | *xanQ_2* | putative transmembrane transport protein | group_521 | membrane protein |
|  |  | *ybcJ* | putative RNA-binding protein | group_533 | putative phage associated protein |
|  |  | *ybcO* | phage associated protein | group_534 | FMN oxidoreductase CC3083 |
|  |  | *yjiA* | CobW-like protein | group_537 | cell division protein |
|  |  | *yusV* | Iron(III) ABC transporter, ATP-binding protein | group_567 | putative transmembrane transport protein |
|  |  | *zraR* | Rsp | group_570 | bacteriophage N4 receptor, outer membrane subunit |
|  |  |  |  | group_575 | putative competence protein ComJ |
|  |  |  |  | group_581 | putative phosphoribosyltransferase |
|  |  |  |  | group_637 | Putative periplasmic protein |
|  |  |  |  | group_639 | PhnO-like protein |
|  |  |  |  | group_657 | phage protein |
|  |  |  |  | group_675 | phage associated protein |
|  |  |  |  | group_687 | phage associated protein |
|  |  |  |  | group_691 | FAD-binding protein |
|  |  |  |  | group_707 | Putative hemoglobin receptor component HpuA |
|  |  |  |  | group_727 | putative metal-dependent phosphoesterase |
|  |  |  |  | group_729 | putative lipoprotein |
|  |  |  |  | group_730 | stress-sensitive restriction system protein |
|  |  |  |  | group_750 | putative CsgG-like lipoprotein |
|  |  |  |  | group_753 | Bacterial capsule synthesis protein PGA_cap |
|  |  |  |  | group_769 | putative transposase |
|  |  |  |  | group_787 | integral membrane protein |
|  |  |  |  | group_795 | PilN |
|  |  |  |  | group_799 | Lipopolysaccharide biosynthesis translocase |
|  |  |  |  | group_814 | NgoPII restriction and modification |
|  |  |  |  | group_825 | integral membrane protein |
|  |  |  |  | group_826 | putative zinc finger/helix-turn-helix protein, YgiT family |
|  |  |  |  | group_827 | Periplasmic protein |
|  |  |  |  | group_833 | FMN-binding protein |
|  |  |  |  | group_836 | putative integral membrane protein |
|  |  |  |  | group_842 | membrane protein |
|  |  |  |  | group_844 | phage associated protein |
|  |  |  |  | group_862 | putative lipoprotein |
|  |  |  |  | group_863 | Cnp1 |
|  |  |  |  | group_894 | integral membrane protein |
|  |  |  |  | group_897 | surface exposed protein |
|  |  |  |  | group_901 | ATP dependent DNA helicase |
|  |  |  |  | group_907 | phage associated protein |
|  |  |  |  | group_909 | putative type I restriction-modification system DNA methylase |
|  |  |  |  | group_915 | lipoprotein |
|  |  |  |  | group_939 | membrane protein |
|  |  |  |  | group_987 | putative oxidoreductase |
|  |  |  |  | group_993 | putative lipoprotein |
|  |  |  |  | group_994 | putative ferredoxin |
|  |  |  |  | group_995 | integral membrane protein |
|  |  |  |  | group_996 | Putative hemolysin |
|  |  |  |  | group_998 | secreted protein |
|  |  |  |  | *hda* | DnaA regulatory inactivator Hda |
|  |  |  |  | *hfq* | host factor-I protein |
|  |  |  |  | *hlyB* | putative ABC transporter |
|  |  |  |  | *hrpA'* | ATP-dependent DNA helicase |
|  |  |  |  | *hsaA* | acyl-CoA dehydrogenase |
|  |  |  |  | *hsdM* | Type I restriction-modification system DNA methylase |
|  |  |  |  | *htrB_2* | lipid A biosynthesis lauroyl acyltransferase |
|  |  |  |  | *infA* | translation initiation factor IF-1 |
|  |  |  |  | *iscA* | HesB/YadR/YfhF family protein |
|  |  |  |  | *ispH* | 4-hydroxy-3-methylbut-2-enyl diphosphate reductase |
|  |  |  |  | *ldcA* | putative muramoyltetrapeptide carboxypeptidase (LD-carboxypeptidase A) |
|  |  |  |  | *leuA* | 2-isopropylmalate synthase |
|  |  |  |  | *leuC* | isopropylmalate isomerase large subunit |
|  |  |  |  | *lex1_2* | LgtE |
|  |  |  |  | *lolA* | LolA protein |
|  |  |  |  | *lplT* | major facilitator family transporter |
|  |  |  |  | *mafA1* | MafA-like protein |
|  |  |  |  | *mdaB* | modulator of drug activity |
|  |  |  |  | *metN* | ABC transporter ATP-binding protein |
|  |  |  |  | *metR* | transcriptional activator MetR |
|  |  |  |  | *metX* | homoserine O-acetyltransferase |
|  |  |  |  | *minD_2* | putative iron sulfur binding protein, Mrp/NBP35 family protein |
|  |  |  |  | *nagZ* | beta-hexosaminidase |
|  |  |  |  | *nsrR* | transcriptional regulator |
|  |  |  |  | *nudF* | ADP-ribose pyrophosphatase |
|  |  |  |  | *nusG* | transcription antitermination protein NusG |
|  |  |  |  | *oatA_1* | trans-acylase |
|  |  |  |  | *pgaC* | glycosyl transferase family protein |
|  |  |  |  | *piiC_5* | outer membrane protein |
|  |  |  |  | *prfB* | peptide chain release factor 2 |
|  |  |  |  | *prmC_2* | methyltransferase domain protein |
|  |  |  |  | *proB* | glutamate 5-kinase |
|  |  |  |  | *pth* | peptidyl-tRNA hydrolase |
|  |  |  |  | *purH* | bifunctional purine biosynthesis protein PurH [includes: phosphoribosylaminoimidazolecarboxamide formyltransferase (AICAR transformylase) and IMP cyclohydrolase (inosinicase; IMP synthetase; ATIC) |
|  |  |  |  | *purP* | permease |
|  |  |  |  | *pyrF* | orotidine 5'-phosphate decarboxylase |
|  |  |  |  | *pyrG* | CTP synthetase |
|  |  |  |  | *qseC* | protein BasS |
|  |  |  |  | *recD* | recombinase D |
|  |  |  |  | *rimN* | putative translation factor |
|  |  |  |  | *rlmB_2* | RNA methyltransferase |
|  |  |  |  | *rluA* | pseudouridine synthase |
|  |  |  |  | *rpiA* | ribose-5-phosphate isomerase A |
|  |  |  |  | *rpmC* | 50S ribosomal protein L29 |
|  |  |  |  | *rpmE2* | 50S ribosomal protein L31 |
|  |  |  |  | *rpoB* | DNA-directed RNA polymerase subunit beta |
|  |  |  |  | *rpsI* | 30S ribosomal protein S9 |
|  |  |  |  | *rpsQ* | 30S ribosomal protein S17 |
|  |  |  |  | *rsmJ* | Ribosomal RNA small subunit methyltransferase J |
|  |  |  |  | *rsuA* | ribosomal small subunit pseudouridine synthase A |
|  |  |  |  | *sucB* | dihydrolipoamide succinyltransferase |
|  |  |  |  | *thiE* | thiamin-phosphate pyrophosphorylase |
|  |  |  |  | *trkG* | transport protein, potassium |
|  |  |  |  | *trmD* | tRNA (guanine-N(1)-)-methyltransferase |
|  |  |  |  | *trmL* | tRNA/rRNA methyltransferase |
|  |  |  |  | *trpF* | N-(5'-phosphoribosyl)anthranilate isomerase |
|  |  |  |  | *ttcA* | C32 tRNA thiolase |
|  |  |  |  | *wcaJ* | PglB protein |
|  |  |  |  | *xanQ_1* | putative transmembrane transport protein |
|  |  |  |  | *xthA* | exodeoxyribonuclease III |
|  |  |  |  | *ybhI* | C4-dicarboxylate transporter |
|  |  |  |  | *ycbX* | iron-sulfur binding protein |
|  |  |  |  | *yccM* | Regulatory protein NosR |
|  |  |  |  | *yccS* | integral membrane protein |
|  |  |  |  | *yceI* | Periplasmic protein |
|  |  |  |  | *yfhA* | ABC transporter permease, enterobactin |
|  |  |  |  | *yhbE* | putative transporter |
|  |  |  |  | *yhbU* | protease |
|  |  |  |  | *yjjK* | ABC transporter ATP-binding protein |
|  |  |  |  | *ypjD* | CcsA-like protein |
|  |  |  |  | *zur* | putative cation uptake regulator |
|  |  |  |  |  |  |

**Table S2.** European Nucleotide Archive (ENA) accession number for *Neisseria gonorrhoeae* isolates included in this study.

| **Study accession** | **Sample accession** | **Secondary sample accession** | **Experiment alias** |
| --- | --- | --- | --- |
| PRJEB4024 | SAMEA2228978 | ERS363132 | SC_EXP_11791_8#3 |
| PRJEB4024 | SAMEA2228979 | ERS363133 | SC_EXP_11791_8#4 |
| PRJEB4024 | SAMEA2228980 | ERS363134 | SC_EXP_11791_8#5 |
| PRJEB4024 | SAMEA2228981 | ERS363135 | SC_EXP_11791_8#6 |
| PRJEB4024 | SAMEA2228982 | ERS363136 | SC_EXP_11791_8#7 |
| PRJEB4024 | SAMEA2228983 | ERS363137 | SC_EXP_11791_8#9 |
| PRJEB4024 | SAMEA2228984 | ERS363138 | SC_EXP_11791_8#10 |
| PRJEB4024 | SAMEA2228985 | ERS363139 | SC_EXP_11791_8#11 |
| PRJEB4024 | SAMEA2228986 | ERS363140 | SC_EXP_11791_8#12 |
| PRJEB4024 | SAMEA2228987 | ERS363141 | SC_EXP_11791_8#13 |
| PRJEB4024 | SAMEA2228988 | ERS363142 | SC_EXP_11791_8#14 |
| PRJEB4024 | SAMEA2228989 | ERS363143 | SC_EXP_11791_8#15 |
| PRJEB4024 | SAMEA2228990 | ERS363144 | SC_EXP_11791_8#16 |
| PRJEB4024 | SAMEA2228991 | ERS363145 | SC_EXP_11791_8#17 |
| PRJEB4024 | SAMEA2228995 | ERS363149 | SC_EXP_11791_8#21 |
| PRJEB4024 | SAMEA2228996 | ERS363150 | SC_EXP_11791_8#22 |
| PRJEB4024 | SAMEA2228998 | ERS363152 | SC_EXP_11791_8#24 |
| PRJEB4024 | SAMEA2228999 | ERS363153 | SC_EXP_11791_8#25 |
| PRJEB4024 | SAMEA2229000 | ERS363154 | SC_EXP_11791_8#26 |
| PRJEB4024 | SAMEA2229001 | ERS363155 | SC_EXP_11791_8#27 |
| PRJEB4024 | SAMEA2229002 | ERS363156 | SC_EXP_11791_8#28 |
| PRJEB4024 | SAMEA2229003 | ERS363157 | SC_EXP_11791_8#29 |
| PRJEB4024 | SAMEA2229004 | ERS363158 | SC_EXP_11791_8#30 |
| PRJEB4024 | SAMEA2229005 | ERS363159 | SC_EXP_11791_8#32 |
| PRJEB4024 | SAMEA2229006 | ERS363160 | SC_EXP_11791_8#33 |
| PRJEB4024 | SAMEA2229007 | ERS363161 | SC_EXP_11791_8#34 |
| PRJEB4024 | SAMEA2229008 | ERS363162 | SC_EXP_11791_8#35 |
| PRJEB4024 | SAMEA2229009 | ERS363163 | SC_EXP_11791_8#36 |
| PRJEB4024 | SAMEA2229010 | ERS363164 | SC_EXP_11791_8#37 |
| PRJEB4024 | SAMEA2229011 | ERS363165 | SC_EXP_11791_8#38 |
| PRJEB4024 | SAMEA2229012 | ERS363166 | SC_EXP_11791_8#39 |
| PRJEB4024 | SAMEA2229013 | ERS363167 | SC_EXP_11791_8#40 |
| PRJEB4024 | SAMEA2229014 | ERS363168 | SC_EXP_11791_8#41 |
| PRJEB4024 | SAMEA2229015 | ERS363169 | SC_EXP_11791_8#42 |
| PRJEB4024 | SAMEA2229017 | ERS363171 | SC_EXP_11791_8#45 |
| PRJEB4024 | SAMEA2229018 | ERS363172 | SC_EXP_11791_8#46 |
| PRJEB4024 | SAMEA2229019 | ERS363173 | SC_EXP_11791_8#47 |
| PRJEB4024 | SAMEA2229020 | ERS363174 | SC_EXP_11791_8#48 |
| PRJEB4024 | SAMEA2229021 | ERS363175 | SC_EXP_11791_8#49 |
| PRJEB4024 | SAMEA2229022 | ERS363176 | SC_EXP_11791_8#50 |
| PRJEB4024 | SAMEA2229023 | ERS363177 | SC_EXP_11791_8#51 |
| PRJEB4024 | SAMEA2229025 | ERS363179 | SC_EXP_11791_8#53 |
| PRJEB4024 | SAMEA2229028 | ERS363182 | SC_EXP_11791_8#56 |
| PRJEB4024 | SAMEA2229029 | ERS363183 | SC_EXP_11791_8#57 |
| PRJEB4024 | SAMEA2229030 | ERS363184 | SC_EXP_11791_8#58 |
| PRJEB4024 | SAMEA2229031 | ERS363185 | SC_EXP_11791_8#59 |
| PRJEB4024 | SAMEA2229032 | ERS363186 | SC_EXP_11791_8#60 |
| PRJEB4024 | SAMEA2229035 | ERS363189 | SC_EXP_11791_8#63 |
| PRJEB4024 | SAMEA2229036 | ERS363190 | SC_EXP_11791_8#64 |
| PRJEB4024 | SAMEA2229037 | ERS363191 | SC_EXP_11791_8#65 |
| PRJEB4024 | SAMEA2229038 | ERS363192 | SC_EXP_11791_8#66 |
| PRJEB4024 | SAMEA2229039 | ERS363193 | SC_EXP_11791_8#67 |
| PRJEB4024 | SAMEA2229040 | ERS363194 | SC_EXP_11791_8#68 |
| PRJEB4024 | SAMEA2229041 | ERS363195 | SC_EXP_11791_8#69 |
| PRJEB4024 | SAMEA2229042 | ERS363196 | SC_EXP_11791_8#70 |
| PRJEB4024 | SAMEA2229043 | ERS363197 | SC_EXP_11791_8#71 |
| PRJEB4024 | SAMEA2229044 | ERS363198 | SC_EXP_11791_8#72 |
| PRJEB4024 | SAMEA2229045 | ERS363199 | SC_EXP_11791_8#73 |
| PRJEB4024 | SAMEA2229046 | ERS363200 | SC_EXP_11791_8#74 |
| PRJEB4024 | SAMEA2229047 | ERS363201 | SC_EXP_11791_8#75 |
| PRJEB4024 | SAMEA2229048 | ERS363202 | SC_EXP_11791_8#76 |
| PRJEB4024 | SAMEA2229049 | ERS363203 | SC_EXP_11791_8#77 |
| PRJEB4024 | SAMEA2229050 | ERS363204 | SC_EXP_11791_8#78 |
| PRJEB4024 | SAMEA2229051 | ERS363205 | SC_EXP_11791_8#79 |
| PRJEB4024 | SAMEA2229052 | ERS363206 | SC_EXP_11791_8#80 |
| PRJEB4024 | SAMEA2229053 | ERS363207 | SC_EXP_11791_8#81 |
| PRJEB4024 | SAMEA2229054 | ERS363208 | SC_EXP_11791_8#82 |
| PRJEB4024 | SAMEA2229055 | ERS363209 | SC_EXP_11791_8#83 |
| PRJEB4024 | SAMEA2229056 | ERS363210 | SC_EXP_11791_8#84 |
| PRJEB4024 | SAMEA2229057 | ERS363211 | SC_EXP_11791_8#85 |
| PRJEB4024 | SAMEA2229058 | ERS363212 | SC_EXP_11791_8#86 |
| PRJEB4024 | SAMEA2229059 | ERS363213 | SC_EXP_11791_8#87 |
| PRJEB4024 | SAMEA2229060 | ERS363214 | SC_EXP_11791_8#88 |
| PRJEB4024 | SAMEA2229061 | ERS363215 | SC_EXP_11791_8#89 |
| PRJEB4024 | SAMEA2229062 | ERS363216 | SC_EXP_11791_8#90 |
| PRJEB4024 | SAMEA2229063 | ERS363217 | SC_EXP_11791_8#91 |
| PRJEB4024 | SAMEA2229064 | ERS363218 | SC_EXP_11791_8#92 |
| PRJEB4024 | SAMEA2229065 | ERS363219 | SC_EXP_11791_8#93 |
| PRJEB4024 | SAMEA2229066 | ERS363220 | SC_EXP_11791_8#94 |
| PRJEB4024 | SAMEA2229067 | ERS363221 | SC_EXP_11791_8#95 |
| PRJEB4024 | SAMEA2229068 | ERS363222 | SC_EXP_11791_8#96 |
| PRJEB4024 | SAMEA2229069 | ERS363223 | SC_EXP_11792_5#1 |
| PRJEB4024 | SAMEA2229070 | ERS363224 | SC_EXP_11792_5#2 |
| PRJEB4024 | SAMEA2229071 | ERS363225 | SC_EXP_11792_5#3 |
| PRJEB4024 | SAMEA2229072 | ERS363226 | SC_EXP_11792_5#4 |
| PRJEB4024 | SAMEA2229073 | ERS363227 | SC_EXP_11792_5#5 |
| PRJEB4024 | SAMEA2229074 | ERS363228 | SC_EXP_11792_5#6 |
| PRJEB4024 | SAMEA2229075 | ERS363229 | SC_EXP_11792_5#7 |
| PRJEB4024 | SAMEA2229076 | ERS363230 | SC_EXP_11792_5#9 |
| PRJEB4024 | SAMEA2229077 | ERS363231 | SC_EXP_11792_5#10 |
| PRJEB4024 | SAMEA2229078 | ERS363232 | SC_EXP_11792_5#11 |
| PRJEB4024 | SAMEA2229079 | ERS363233 | SC_EXP_11792_5#12 |
| PRJEB4024 | SAMEA2229080 | ERS363234 | SC_EXP_11792_5#13 |
| PRJEB4024 | SAMEA2229081 | ERS363235 | SC_EXP_11792_5#14 |
| PRJEB4024 | SAMEA2229082 | ERS363236 | SC_EXP_11792_5#15 |
| PRJEB4024 | SAMEA2229083 | ERS363237 | SC_EXP_11792_5#16 |
| PRJEB4024 | SAMEA2229084 | ERS363238 | SC_EXP_11792_5#17 |
| PRJEB4024 | SAMEA2229085 | ERS363239 | SC_EXP_11792_5#18 |
| PRJEB4024 | SAMEA2229086 | ERS363240 | SC_EXP_11792_5#19 |
| PRJEB4024 | SAMEA2229087 | ERS363241 | SC_EXP_11792_5#20 |
| PRJEB4024 | SAMEA2229088 | ERS363242 | SC_EXP_11792_5#21 |
| PRJEB4024 | SAMEA2229089 | ERS363243 | SC_EXP_11792_5#22 |
| PRJEB4024 | SAMEA2229090 | ERS363244 | SC_EXP_11792_5#23 |
| PRJEB4024 | SAMEA2229091 | ERS363245 | SC_EXP_11792_5#24 |
| PRJEB4024 | SAMEA2229092 | ERS363246 | SC_EXP_11792_5#25 |
| PRJEB4024 | SAMEA2229093 | ERS363247 | SC_EXP_11792_5#26 |
| PRJEB4024 | SAMEA2229094 | ERS363248 | SC_EXP_11792_5#27 |
| PRJEB4024 | SAMEA2229095 | ERS363249 | SC_EXP_11792_5#28 |
| PRJEB4024 | SAMEA2229096 | ERS363250 | SC_EXP_11792_5#29 |
| PRJEB4024 | SAMEA2229097 | ERS363251 | SC_EXP_11792_5#30 |
| PRJEB4024 | SAMEA2229098 | ERS363252 | SC_EXP_11792_5#32 |
| PRJEB4024 | SAMEA2229099 | ERS363253 | SC_EXP_11792_5#33 |
| PRJEB4024 | SAMEA2229100 | ERS363254 | SC_EXP_11792_5#34 |
| PRJEB4024 | SAMEA2229101 | ERS363255 | SC_EXP_11792_5#35 |
| PRJEB4024 | SAMEA2229102 | ERS363256 | SC_EXP_11792_5#36 |
| PRJEB4024 | SAMEA2229103 | ERS363257 | SC_EXP_11792_5#37 |
| PRJEB4024 | SAMEA2229104 | ERS363258 | SC_EXP_11792_5#38 |
| PRJEB4024 | SAMEA2229105 | ERS363259 | SC_EXP_11792_5#39 |
| PRJEB4024 | SAMEA2229106 | ERS363260 | SC_EXP_11792_5#40 |
| PRJEB4024 | SAMEA2229107 | ERS363261 | SC_EXP_11792_5#41 |
| PRJEB4024 | SAMEA2229108 | ERS363262 | SC_EXP_11792_5#42 |
| PRJEB4024 | SAMEA2229109 | ERS363263 | SC_EXP_11792_5#44 |
| PRJEB4024 | SAMEA2229110 | ERS363264 | SC_EXP_11792_5#45 |
| PRJEB4024 | SAMEA2229111 | ERS363265 | SC_EXP_11792_5#46 |
| PRJEB4024 | SAMEA2229112 | ERS363266 | SC_EXP_11792_5#47 |
| PRJEB4024 | SAMEA2229113 | ERS363267 | SC_EXP_11792_5#48 |
| PRJEB4024 | SAMEA2229114 | ERS363268 | SC_EXP_11792_5#49 |
| PRJEB4024 | SAMEA2229115 | ERS363269 | SC_EXP_11792_5#50 |
| PRJEB4024 | SAMEA2229116 | ERS363270 | SC_EXP_11792_5#51 |
| PRJEB4024 | SAMEA2298446 | ERS390904 | SC_EXP_12273_1#1 |
| PRJEB4024 | SAMEA2298447 | ERS390905 | SC_EXP_12273_1#2 |
| PRJEB4024 | SAMEA2298448 | ERS390906 | SC_EXP_12273_1#3 |
| PRJEB4024 | SAMEA2298449 | ERS390907 | SC_EXP_12273_1#4 |
| PRJEB4024 | SAMEA2298450 | ERS390908 | SC_EXP_12273_1#5 |
| PRJEB4024 | SAMEA2298451 | ERS390909 | SC_EXP_12273_1#6 |
| PRJEB4024 | SAMEA2298452 | ERS390910 | SC_EXP_12273_1#7 |
| PRJEB4024 | SAMEA2298453 | ERS390911 | SC_EXP_12273_1#8 |
| PRJEB4024 | SAMEA2298454 | ERS390912 | SC_EXP_12273_1#9 |
| PRJEB4024 | SAMEA2298455 | ERS390913 | SC_EXP_12273_1#10 |
| PRJEB4024 | SAMEA2298456 | ERS390914 | SC_EXP_12273_1#11 |
| PRJEB4024 | SAMEA2298457 | ERS390915 | SC_EXP_12273_1#12 |
| PRJEB4024 | SAMEA2298458 | ERS390916 | SC_EXP_12273_1#13 |
| PRJEB4024 | SAMEA2298459 | ERS390917 | SC_EXP_12273_1#14 |
| PRJEB4024 | SAMEA2298460 | ERS390918 | SC_EXP_12273_1#15 |
| PRJEB4024 | SAMEA2298461 | ERS390919 | SC_EXP_12273_1#16 |
| PRJEB4024 | SAMEA2298462 | ERS390920 | SC_EXP_12273_1#17 |
| PRJEB4024 | SAMEA2298463 | ERS390921 | SC_EXP_12273_1#18 |
| PRJEB4024 | SAMEA2298464 | ERS390922 | SC_EXP_12273_1#19 |
| PRJEB4024 | SAMEA2298465 | ERS390923 | SC_EXP_12273_1#20 |
| PRJEB4024 | SAMEA2298466 | ERS390924 | SC_EXP_12273_1#21 |
| PRJEB4024 | SAMEA2298467 | ERS390925 | SC_EXP_12273_1#22 |
| PRJEB4024 | SAMEA2298468 | ERS390926 | SC_EXP_12273_1#23 |
| PRJEB4024 | SAMEA2298469 | ERS390927 | SC_EXP_12273_1#24 |
| PRJEB4024 | SAMEA2298470 | ERS390928 | SC_EXP_12273_1#25 |
| PRJEB4024 | SAMEA2298471 | ERS390929 | SC_EXP_12273_1#26 |
| PRJEB4024 | SAMEA2298472 | ERS390930 | SC_EXP_12273_1#27 |
| PRJEB4024 | SAMEA2298473 | ERS390931 | SC_EXP_12273_1#28 |
| PRJEB4024 | SAMEA2298474 | ERS390932 | SC_EXP_12273_1#29 |
| PRJEB4024 | SAMEA2298475 | ERS390933 | SC_EXP_12273_1#30 |
| PRJEB4024 | SAMEA2298476 | ERS390934 | SC_EXP_12273_1#31 |
| PRJEB4024 | SAMEA2298477 | ERS390935 | SC_EXP_12273_1#32 |
| PRJEB4024 | SAMEA2298478 | ERS390936 | SC_EXP_12273_1#33 |
| PRJEB4024 | SAMEA2298479 | ERS390937 | SC_EXP_12273_1#34 |
| PRJEB4024 | SAMEA2298480 | ERS390938 | SC_EXP_12273_1#35 |
| PRJEB4024 | SAMEA2298481 | ERS390939 | SC_EXP_12273_1#36 |
| PRJEB4024 | SAMEA2298482 | ERS390940 | SC_EXP_12273_1#37 |
| PRJEB4024 | SAMEA2298483 | ERS390941 | SC_EXP_12273_1#38 |
| PRJEB4024 | SAMEA2298484 | ERS390942 | SC_EXP_12273_1#39 |
| PRJEB4024 | SAMEA2298485 | ERS390943 | SC_EXP_12273_1#40 |
| PRJEB4024 | SAMEA2298486 | ERS390944 | SC_EXP_12273_1#41 |
| PRJEB4024 | SAMEA2298487 | ERS390945 | SC_EXP_12273_1#42 |
| PRJEB4024 | SAMEA2298488 | ERS390946 | SC_EXP_12273_1#43 |
| PRJEB4024 | SAMEA2298489 | ERS390947 | SC_EXP_12273_1#44 |
| PRJEB4024 | SAMEA2298490 | ERS390948 | SC_EXP_12273_1#45 |
| PRJEB4024 | SAMEA2298491 | ERS390949 | SC_EXP_12273_1#46 |
| PRJEB4024 | SAMEA2298492 | ERS390950 | SC_EXP_12273_1#47 |
| PRJEB4024 | SAMEA2298493 | ERS390951 | SC_EXP_12273_1#48 |
| PRJEB4024 | SAMEA2298494 | ERS390952 | SC_EXP_12273_1#49 |
| PRJEB4024 | SAMEA2298495 | ERS390953 | SC_EXP_12273_1#50 |
| PRJEB4024 | SAMEA2298496 | ERS390954 | SC_EXP_12273_1#51 |
| PRJEB4024 | SAMEA2298497 | ERS390955 | SC_EXP_12273_1#52 |
| PRJEB4024 | SAMEA2298498 | ERS390956 | SC_EXP_12273_1#53 |
| PRJEB4024 | SAMEA2298499 | ERS390957 | SC_EXP_12273_1#54 |
| PRJEB4024 | SAMEA2298500 | ERS390958 | SC_EXP_12273_1#55 |
| PRJEB4024 | SAMEA2298501 | ERS390959 | SC_EXP_12273_1#56 |
| PRJEB4024 | SAMEA2298502 | ERS390960 | SC_EXP_12273_1#57 |
| PRJEB4024 | SAMEA2298503 | ERS390961 | SC_EXP_12273_1#58 |
| PRJEB4024 | SAMEA2298504 | ERS390962 | SC_EXP_12273_1#59 |
| PRJEB4024 | SAMEA2298505 | ERS390963 | SC_EXP_12273_1#60 |
| PRJEB4024 | SAMEA2298506 | ERS390964 | SC_EXP_12273_1#61 |
| PRJEB4024 | SAMEA2298507 | ERS390965 | SC_EXP_12273_1#62 |
| PRJEB4024 | SAMEA2738422 | ERS538093 | SC_EXP_14936_7#1 |
| PRJEB4024 | SAMEA2738424 | ERS538095 | SC_EXP_14936_7#3 |
| PRJEB4024 | SAMEA2738426 | ERS538097 | SC_EXP_14936_7#5 |
| PRJEB4024 | SAMEA2738436 | ERS538107 | SC_EXP_14936_7#15 |
| PRJEB4024 | SAMEA2738438 | ERS538109 | SC_EXP_14936_7#17 |
| PRJEB4024 | SAMEA2738439 | ERS538110 | SC_EXP_14936_7#18 |
| PRJEB4024 | SAMEA2738441 | ERS538112 | SC_EXP_14936_7#20 |
| PRJEB4024 | SAMEA2738442 | ERS538113 | SC_EXP_14936_7#21 |
| PRJEB4024 | SAMEA2738446 | ERS538117 | SC_EXP_14936_7#25 |
| PRJEB4024 | SAMEA2738447 | ERS538118 | SC_EXP_14936_7#26 |
| PRJEB4024 | SAMEA2738448 | ERS538119 | SC_EXP_14936_7#27 |
| PRJEB4024 | SAMEA2738450 | ERS538121 | SC_EXP_14936_7#29 |
| PRJEB4024 | SAMEA2738452 | ERS538123 | SC_EXP_14936_7#31 |
| PRJEB4024 | SAMEA2738454 | ERS538125 | SC_EXP_14936_7#33 |
| PRJEB4024 | SAMEA2738462 | ERS538133 | SC_EXP_14936_7#41 |
| PRJEB4024 | SAMEA2738465 | ERS538136 | SC_EXP_14936_7#44 |
| PRJEB4024 | SAMEA2738470 | ERS538141 | SC_EXP_14936_7#49 |
| PRJEB4024 | SAMEA2738475 | ERS538146 | SC_EXP_14936_7#54 |
| PRJEB4024 | SAMEA2738476 | ERS538147 | SC_EXP_14936_7#55 |
| PRJEB4024 | SAMEA2738478 | ERS538149 | SC_EXP_14936_7#57 |
| PRJEB4024 | SAMEA2738479 | ERS538150 | SC_EXP_14936_7#58 |
| PRJEB4024 | SAMEA2738484 | ERS538155 | SC_EXP_14936_7#63 |
| PRJEB4024 | SAMEA2738487 | ERS538158 | SC_EXP_14936_7#66 |
| PRJEB4024 | SAMEA2738490 | ERS538161 | SC_EXP_14936_7#69 |
| PRJEB4024 | SAMEA2738494 | ERS538165 | SC_EXP_14936_7#73 |
| PRJEB4024 | SAMEA2738496 | ERS538167 | SC_EXP_14936_7#75 |
| PRJEB4024 | SAMEA2738497 | ERS538168 | SC_EXP_14936_7#76 |
| PRJEB4024 | SAMEA2738502 | ERS538173 | SC_EXP_14936_7#81 |
| PRJEB4024 | SAMEA2738503 | ERS538174 | SC_EXP_14936_7#82 |
| PRJEB4024 | SAMEA2738504 | ERS538175 | SC_EXP_14936_7#83 |
| PRJEB4024 | SAMEA2738505 | ERS538176 | SC_EXP_14936_7#84 |
| PRJEB4024 | SAMEA2738509 | ERS538180 | SC_EXP_14936_7#88 |
| PRJEB4024 | SAMEA2738510 | ERS538181 | SC_EXP_14936_7#89 |
| PRJEB4024 | SAMEA2738512 | ERS538183 | SC_EXP_14936_7#91 |
| PRJEB4024 | SAMEA2738513 | ERS538184 | SC_EXP_14936_7#92 |
| PRJEB4024 | SAMEA2738514 | ERS538185 | SC_EXP_14936_7#93 |
| PRJEB4024 | SAMEA2738515 | ERS538186 | SC_EXP_14936_7#94 |
| PRJEB4024 | SAMEA2738416 | ERS538087 | SC_EXP_14937_1#2 |
| PRJEB4024 | SAMEA2738417 | ERS538088 | SC_EXP_14937_1#3 |
| PRJEB4024 | SAMEA2738418 | ERS538089 | SC_EXP_14937_1#4 |
